# A centromere-derived retroelement RNA localizes in *cis* and is a core element of the transcriptional landscape of *Drosophila* centromeres

**DOI:** 10.1101/2024.01.14.574223

**Published:** 2024-01-16

**Authors:** B Santinello, R Sun, A Amjad, SJ Hoyt, L Ouyang, C Courret, R Drennan, L Leo, AM Larracuente, LM Core, RJ O’Neill, BG Mellone

**Affiliations:** 1 Department of Molecular and Cell Biology, University of Connecticut, Storrs, CT, US; 2 Department of Biology, University of Rochester, Rochester, NY, US; 3 Dipartimento di Biologia e Biotecnologie “Charles Darwin”, “Sapienza” University of Rome, 00185 Rome, Italy; 4 Present address: RNA editing Lab, Onco-Haematology Department, Genetics and Epigenetics of Pediatric Cancers, Bambino Gesù Children Hospital, IRCCS, 00146 Rome, Italy; 5 Institute for Systems Genomics, University of Connecticut, Storrs CT, US; 6 Department of Genetics and Genome Sciences, UConn Health, Farmington, CT, US

## Abstract

Centromeres are essential chromosomal landmarks that dictate the point of attachment between chromosomes and spindle microtubules during cell division. The stable transmission of the centromere site through generations is ensured by a unique chromatin containing the histone H3 variant CENP-A. Previous studies have highlighted the impact of transcription on promoting CENP-A deposition. However, the specific sequences undergoing this transcription and their contribution to centromere function in metazoan systems remain elusive. In this study, we unveil the centromeric transcriptional landscape and explore its correlation with CENP-A in *D. melanogaster*, currently the only *in vivo* model with assembled centromeres. We find that the centromere-enriched retroelement *G2/Jockey-3* (hereafter referred to as *Jockey-3*) is a major driver of centromere transcription, producing RNAs that localize to all mitotic centromeres, with the Y centromere showing the most transcription. Taking advantage of the polymorphism of *Jockey-3*, we show that these RNAs remain associated with their cognate DNA sequences in *cis*. Using a LacI/lacO system to generate *de novo* centromeres, we find that *Jockey-3* transcripts do not localize to ectopic sites, suggesting they are unlikely to function as non-coding RNAs with a structural role at centromeres. At *de novo* centromeres on the lacO array, the presence of CENP-A augments the detection of exogenous lacO-derived transcripts specifically in metaphase. We propose that *Jockey-3* contributes to the epigenetic maintenance of the centromere by promoting chromatin transcription, while inserting in a region that permits its continuous transmission. Given the conservation of retroelements as centromere components across taxa, our findings have broad implications in understanding this widespread association.

## Introduction

Genome partitioning during cell division is dependent on specialized chromosomal structures known as centromeres, which mediate kinetochore assembly. This process is crucial for establishing robust connections between chromosomes and spindle microtubules, essential for the precise segregation of chromosomes. Centromeric chromatin is marked by the presence of nucleosomes containing the histone H3 variant CENP-A (also known as Cid *Drosophila Ref* )(Henikoff et al., 2000; Palmer et al., 1987), which initiates the recruitment of additional centromeric and kinetochore proteins (McKinley and Cheeseman, 2016). Centromeres are paradoxical in that they play a highly conserved function across eukaryotes yet are amongst the most rapidly evolving regions of genomes. Centromeres are also dynamic – they can reposition in individuals (neocentromeres) (Marshall et al., 2008) and become fixed in a population (evolutionary new centromeres)(Ventura et al., 2007). Despite being able to reposition, centromeres are typically associated with large highly repetitive sequences whose role in centromere identity remains elusive.

Transcripts emanating from centromeres have been observed in a myriad of systems, including budding yeast (Hedouin et al., 2022; Ohkuni and Kitagawa, 2011), human cells (Bury et al., 2020; Hoyt et al., 2022; McNulty et al., 2017; Quenet and Dalal, 2014), frog egg extracts (Blower, 2016; Grenfell et al., 2016), maize (Topp et al., 2004) and marsupials (Carone et al., 2013). Transcription at centromeres has been shown to be coupled to *de novo* centromere formation (Chen et al., 2015) and neocentromere formation in humans (Chueh et al., 2009; Murillo-Pineda et al., 2021; Naughton et al., 2022). In addition, centromeric transcription is critical for programmed histone exchange in *S. pombe* (Wu et al., 2022), for the stabilization of newly formed CENP-A nucleosomes in *Drosophila* cells (Bobkov et al., 2018), and for HAC formation (Cardinale et al., 2009). These studies suggest that centromeric DNA may contribute to centromere identity through its ability to be transcribed. Other studies have also implicated a role for centromere-derived transcripts as noncoding RNAs important for centromere integrity (Blower, 2016; Carone et al., 2013; McNulty et al., 2017; Quenet and Dalal, 2014; Topp et al., 2004). Indeed, in some cases centromeric transcripts have been detected associated with centromeric proteins (e.g. (Blower, 2016; Grenfell et al., 2016; McNulty et al., 2017), suggesting a role beyond being a byproduct of transcription. However, it remains unresolved whether the interaction with centromere proteins is sequence-specific. Furthermore, both the functional impact of these RNAs, as well as the extent of their prevalence across different systems, are still not fully understood.

Consistent with the existence of centromeric transcripts, elongating RNA polymerase II accumulates at mitotic centromeres in *Drosophila* S2 cells (Bobkov et al., 2018; Rosic et al., 2014) and nascent transcription can be detected at the centromere of *Drosophila* S2 cells in mitosis and G1 (Bobkov et al., 2018). However, the RNA products of such centromeric transcription are unknown. A previous study analyzed the role of a non-coding RNA produced by a satellite of the 1.688 family in *Drosophila*, showing that its depletion affects accurate chromosome segregation and centromere integrity (Rosic et al., 2014). However, the largest block of this satellite is located within pericentric heterochromatin on the X (Chang et al., 2019) and its RNA product does not localize to centromeres (Bobkov et al., 2018). Therefore, its contributions to centromere segregation accuracy might be unrelated to centromeric defects.

The centromeres of *Drosophila melanogaster* have been recently annotated, providing a unique opportunity to directly analyze transcripts associated with centromeres. *Drosophila* has five chromosomes (X; Y; 2; 3; and 4), each harboring a unique centromere differing in repeat composition and organization. The centromeres are composed of islands of complex repeats enriched in retroelements embedded in large arrays of simple satellites. CENP-A occupies primarily the islands, which are between 101–171-kb, extending only partially to the flanking satellites. All of the repeats present at *Drosophila* centromeres are also present elsewhere in the genome, yet a subset of retroelements are enriched at centromeres (Chang et al., 2019). Only one element, the non-LTR retroelement *G2/Jockey-3* (henceforth *Jockey-3*), is shared between all centromeres and is conserved at the centromeres of *D. simulans*, a species that diverged from *D. melanogaster* 2.5 million years ago (Garrigan et al., 2012) and that displays highly divergent centromeric satellites (Jagannathan et al., 2017). Retroelements are conserved centromere-associated elements across taxa. These elements could contribute to centromere function in two ways: either by facilitating localized transcription to promote CENP-A incorporation (Bobkov et al., 2018; Chan et al., 2012; Chan and Wong, 2012; Chen et al., 2015; Choi et al., 2011; Choi et al., 2012; Mellone and Fachinetti, 2021) or by generating transcripts with non-coding roles in maintaining centromere integrity (Blower, 2016; Grenfell et al., 2016; Klein and O’Neill, 2018; McNulty et al., 2017).

Here, we investigate the expression and RNA localization of the conserved centromere-enriched retroelement *Jockey-3*. Nascent transcription profiling and total RNA-seq in *Drosophila* embryos show that centromeric copies of *Jockey-3* are actively transcribed. Using single-molecule RNA FISH combined with immunofluorescence for the centromere protein CENP-C, we show that, during mitosis, *Jockey-3* RNA transcripts localize primarily to centromeres and remain associated with their locus of origin in *cis*. We also show that CENP-A chromatin occupancy is strongly correlated with nascent transcription at both centromeric and non-centromeric full-length *Jockey-3* copies. *De novo* centromere formation *in vivo* using the LacI/lacO tethering system results in the accumulation of lacO transcripts at the *de novo* centromere, suggesting that CENP-A chromatin formation is coupled with transcription *in vivo*. Our work supports a model whereby an active retroelement contributes to CENP-A chromatin maintenance through its transcription. This could be a mechanism for retroelements to ‘hide’ from transcriptional silencing defense mechanisms and ensure their continued propagation.

## Results

### The transcriptional landscape of *Drosophila* centromeres

**Figure 1.**
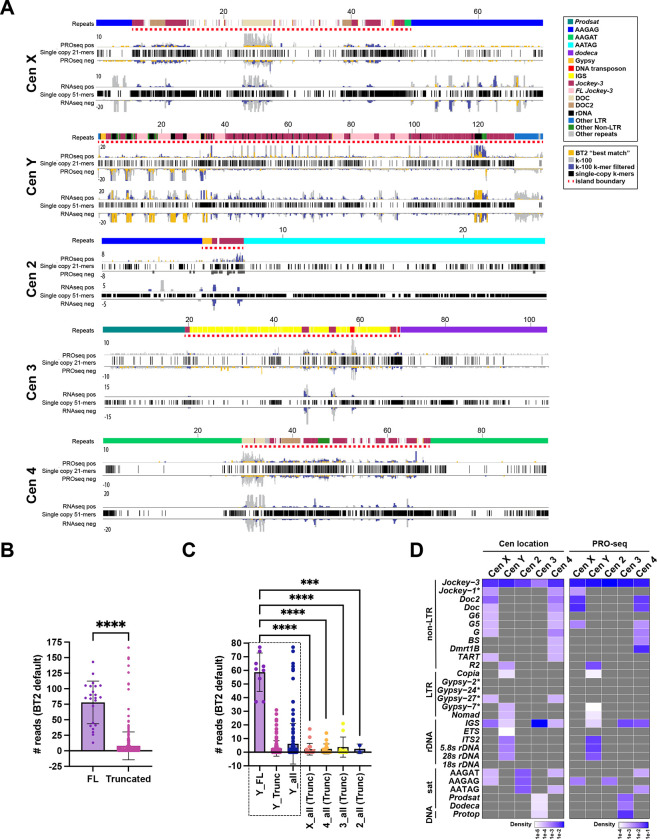
*Jockey-3* is a major driver of centromeric nascent transcription **A** PRO-seq, RNA-seq signals for 0–12h embryos across all *D. melanogaster* centromeres. Top track shows sense, bottom, antisense. Tracks show read coverage with three mapping methods: Bowtie 2 default “best match” (“lower bounds”; yellow), over-fit (“upper bounds”; gray) and a filtered over-fit (“intermediate bounds”; blue). For PRO-seq we used Bowtie k-100 for over-fit, and Bowtie k-100 unique 21-mer filtered for intermediate bounds. For RNA-seq we used Bowtie2 k-100 for over-fit and Bowtie2 k-100 unique 51-mer filtered for intermediate bounds. Repeat annotation is shown on top (see legend for details), with unique 21 and 51-mers (black) used for the filtering shown below. The k-mer tracks illustrate the regions that lack sequence specificity and are therefore most prone to read loss through k-mer filtering. Coordinates shown are kilobases. The boundaries of centromere islands are demarcated by a red dashed line. **B** PRO-seq read density scatter boxplot comparison between full-length and truncated (minus three outliers) *Jockey-3* copies, regardless of genome location. Mapping was done with Bowtie 2 default “best match” using paired-end reads. An unpaired t-test determined a statistically significant difference (****; p < 0.0001; Student’s t-test). Standard deviation error bars are shown. **C** PRO-seq read density scatter-boxplot comparisons of centromeric *Jockey-3* copies split by chromosome and whether they are full-length vs. truncated. Since chromosome Y includes both full-length and truncated copies, a third bar was included encompassing all copies; all three bars are indicated by a dashed box. Mapping was performed with Bowtie 2 default “best match” using paired-end reads. Standard deviation error bars are shown. **D** Leg, density plot of all repetitive elements on each candidate centromere contig grouped by type as in Chang et al (non-LTR retroelements, LTR retroelements, rDNA-related sequences, simple satellites, and DNA transposon) using an updated genome annotation from Hemmer et al. An * indicates annotations based on similarity to retroelements in other *Drosophila* species: *Jockey-1* and *Gypsy-2* are from *D. simulans*, *Gypsy-24* and *Gypsy-27* are from *D. yakuba*, and *Gypsy-7* is from *D. sechellia*. Right, density plots showing PRO-seq reads (k-100 filtered) for a given repeat (see label from C) normalized by the total number of reads mapping to each contig. Density scale is shown in blue. Gray indicates zero copies/reads for a given repeat.

Transcription of centromeric DNA has been implicated in both a sequence-independent manner and through the action of specific transcripts (Grenfell et al., 2017; Mellone and Fachinetti, 2021; Perea-Resa and Blower, 2018; Rosic and Erhardt, 2016). In *Drosophila*, only a few known satellite transcripts have been identified (Bobkov et al., 2018; Mills et al., 2019; Rosic et al., 2014; Wei et al., 2021), but these are either pericentric or not derived from the sequences most highly associated with CENP-A (Chang et al., 2019). The availability of annotated centromeres for the *Drosophila* laboratory strain iso-1 and the discovery that these centromeres contain retroelements (Chang et al., 2019) presents a unique opportunity to examine transcription across these previously unresolved regions of the genome and explore the correlation with CENP-A occupancy. To identify nascent transcripts, we generated libraries for Precision Nuclear Run-On sequencing (PRO-seq), which detects nascent transcription from RNA polymerase with nucleotide resolution (Mahat et al., 2016) from 0–12h old embryos and 3rd instar larval brains. We also generated RNA-seq libraries for the same samples, providing a catalog of stable transcripts. Plotting our PRO-seq data for all genes showed the expected transcriptional profile with a peak at the 5’ of genes, confirming successful capture of elongating RNA polymerase (Fig. S1). Since none of the repeats found at the centromeres are unique to these regions and PRO-seq and RNA-seq generate short-read data, nascent transcripts identified by PRO-seq did not map uniquely to the centromeres using standard mapping methods. To overcome this limitation and determine if any nascent transcripts emanate from centromeric sequences, we adapted a mapping-dependent method recently developed for the human repeats transcriptome (Hoyt et al., 2022) to our *Drosophila* datasets. For each dataset, Bowtie 2 default “best match” reports a single alignment for each read providing locus-level transcription profiles (lower bounds); unfiltered Bowtie/Bowtie 2 k-100 mapping reports up to 100 mapped loci for each read, providing over-fitted and locus-level transcriptional profiles (upper bounds); and single copy k-mer filtering applied to Bowtie k-100 with 21-mers for PRO-seq and 51-mers applied to Bowtie2 k-100 for RNA-seq data reveals the intermediate bounds of locus-level transcription (Fig. 1A). This k-mer filtering requires a given read alignment to overlap an entire single copy k-mer in the assembly in order to be retained. Together, these different approaches provide a more complete representation of the true transcriptional landscape of centromeres.

We observe nascent transcription at all centromeres, particularly within the islands (Fig. 1A). Based on our statistical tests, *Jockey-3* nascent transcripts emerge primarily from full-length *Jockey-3* elements (Fig. 1B, Fig. S2; Table S1), 9/23 of which are within the Y centromere while the rest (14/23) are non-centromeric (Table 1). Both centromeric and non-centromeric truncated *Jockey-3* elements are transcribed (Table S1), suggesting that the putative promoter at the 5’ end (Hemmer et al., 2023) is not required for *Jockey-3* transcription. When we compared the number of *Jockey-3* reads mapping to each of the centromeres, classified based on whether they are full-length or truncated, we observed significantly more reads coming from full-length *Jockey-3* insertions within the Y centromere compared to all others (Fig. 1C).

Similarly to nascent RNA data, RNA-seq profiles from embryos reveal the presence of transcripts predominantly mapping to the islands, with low levels of satellite transcripts, with the notable exception of AAGAG on the X centromere, which shows more expression in this dataset (Fig. 1A). PRO-seq from larval brains (Fig. S3), as well as from 0–4h and 4–8h old embryos (data not shown) showed very similar transcriptional profiles. In contrast, RNA-seq profiles from larval brains showed more transcripts mapping to flanking satellites compared to that observed in the embryos datasets (Fig. S3).

To determine more quantitatively which centromere-associated repeats are transcribed, we generated read count plots for each of the repeats found within the centromere contigs. We recreated a density plot of all repetitive elements as in (Chang et al., 2019) using an updated genome annotation (Hemmer, 2023) to show how many copies of each repeat are present within each of the centromere contigs (Fig. 1D-leg plot). We then generated a density heat map for the PRO-seq 0–12h embryos dataset, which displays the total read count for each repeat normalized by the total reads mapping to that contig. This heat map shows that *Jockey-3* is highly expressed at all centromeres (Fig. 1D-right plot and Table S2). Several repeats show background levels of transcription (e.g. *Copia* and *Gypsy-7*), emphasizing that nascent transcription at the centromere occurs primarily at a subset of elements. Collectively, these analyses show that the *Drosophila* centromeres are actively transcribed and that *Jockey-3* in particular contributes significantly to the overall transcription occurring in these regions.

### *Jockey-3* transcripts localize to metaphase centromeres

**Figure 2.**
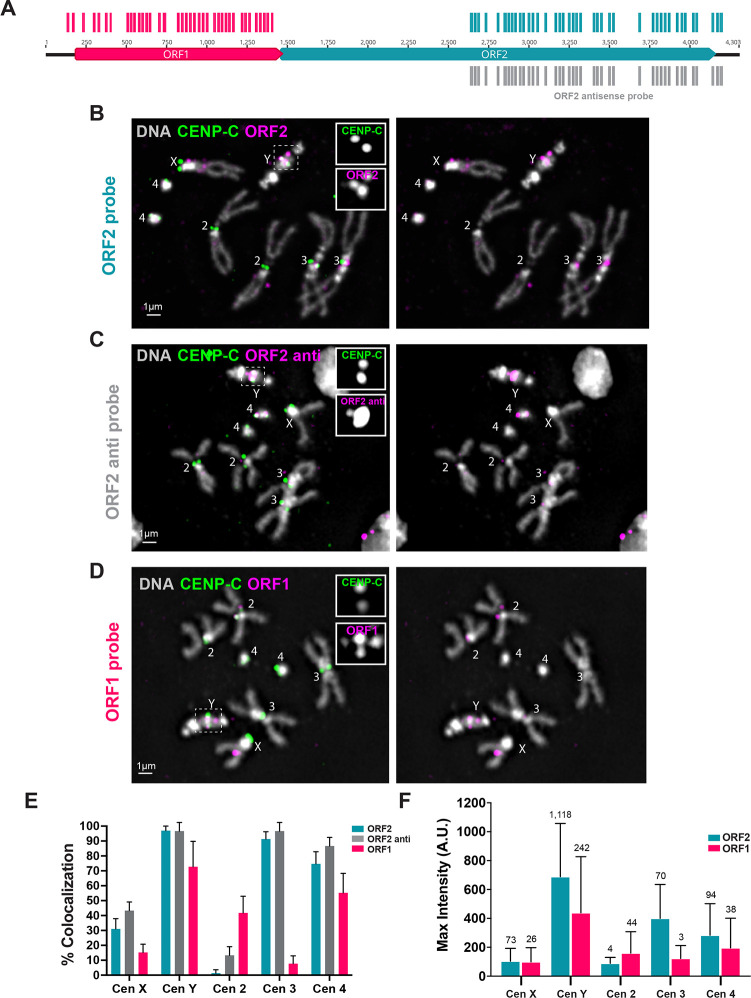
*Jockey-3* transcripts localize to metaphase chromosomes **A** Diagram of *Jockey-3* showing base-pair position, predicted protein domains, and coverage of ORF1 (magents) and ORF2 (teal) probe sets. **B-D** Representative iso-1 male larval brain metaphase spreads. Chromosomes are stained with DAPI (magenta), RNA-FISH for *Jockey-3* ORF2 (B), ORF2 sense (C), and ORF1 (D) probes and IF for CENP-C (green). The images on the leg show the merged channels and a grayscale 1.5x zoom inset for the Y centromere. The images on the right show DAPI and RNA FISH signals. **E** Graph of the percent of mitotic chromosomes showing colocalization between CENP-C and *Jockey-3* RNA FISH signal. ORF2 (N=3 brains, n=83 spreads), ORF2 anti (N=3 brains, n=28 spreads), and ORF1 (N=4 brains, n=69 spreads). **F** Fluorescence intensity plot of centromeric *Jockey-3* RNA FISH signal. ORF2 probe (N= 1 brain, n=30 spreads) and ORF1 (N=1 brain, n=30 spreads). Values above plotted data indicate the number of hits predicted to have complementarity with the corresponding probe set.

*Jockey-3* is the only element that is transcribed at all five *Drosophila* centromeres (Fig. 1C). To examine the subcellular localization of *Jockey-3* transcripts in *D. melanogaster*, we designed strand-specific probes for single-molecule RNA Fluorescence *In Situ* Hybridization (smRNA FISH, henceforth RNA-FISH); one set detects sense transcripts targeting the 5’ region of *Jockey-3*, spanning ORF1, and the other targets the 3’ region, spanning the reverse-transcriptase domain within ORF2 (referred to as ORF1 and ORF2 probes; Fig. 2A). We also generated a reverse-complement set of the ORF2 probe to detect antisense transcripts (ORF2 anti). Each of the probe sets is made up of individual oligos that target both centromeric and non-centromeric *Jockey-3* (ORF1 = 44 oligos; ORF2 = 45 oligos). Several *Jockey-3* insertions across the genome are targeted by five or more probes, and are thus expected to produce RNA-FISH signal if sufficiently expressed, but centromere contigs are the regions targeted the most because 63% of *Jockey-3* copies are centromeric ((Chang et al., 2019); Table 2 and Table S3). Specifically, the ORF2 probe is expected to target primarily *Jockey-3* copies on centromere X, Y, 3, and 4, while the ORF1 probe is expected to target those from centromere X, Y, 2, and 4.

We combined RNA-FISH for *Jockey-3* with immunofluorescence (IF; RNA-FISH/IF) for the centromere protein CENP-C which, unlike CENP-A, is retained on acid-fixed metaphase spreads from larval brain squashes. As a positive control for RNA-FISH, we used a smRNA-FISH probe targeting the *Rox1* non-coding RNA (ncRNA), which coats the X chromosome in males (Meller et al., 1997), Figure S4). We observed transcripts labeled by the ORF2 probe co-localizing with CENP-C at the X, Y, 3rd, and 4th centromeres (Fig. 2B, E and S5), consistent with where these probes sequences map in the assembly (Table 2 and Table S3). We also observed co-localization of ORF2 antisense *Jockey-3* transcripts with CENP-C at the same centromeres (Fig. 2C, E and Fig. S5), indicating the simultaneous presence of both sense and antisense transcripts also shown by our transcript analyses (Fig. 1). Transcripts labeled by the ORF1 probe co-localized with centromeres X, Y, 2, and 4 (Fig. 2D, E and S5), again consistent with our predictions based on our mapping data (Table 2 and Table S3).

The Y centromere is the only centromere containing full-length copies of *Jockey-3* and these show the highest levels of nascent transcription compared to other centromeres (Fig. 1C); thus it is not surprising that the Y displays co-localization between CENP-C and all three probe sets most consistently. In contrast, other chromosomes display more variability (Fig. 2E and S5). In general, the frequency with which we observe co-localization between *Jockey-3* transcripts and CENP-C correlates with the number of probes targeting *Jockey-3* at each particular centromere, with centromere Y being targeted by the most probes overall due to this centromere containing 197/329 total *Jockey-3* copies in the genome ((Chang et al., 2019); Figure 2E, Table 1–2 and Table S3). Fluorescence intensity measurements for individual mitotic centromeres followed the same trend, with stronger signal detected on the Y (Fig. 2F). All five centromeres– including centromere 2, which contains only two *Jockey-3* fragments next to one another– show colocalization with at least one *Jockey-3* probe set. These findings confirm that truncated as well as full-length centromeric *Jockey-3* copies are active, consistent with our transcriptional profiles (Fig. 1). We also confirmed the localization of *Jockey-3* transcripts at metaphase centromeres in mitotic cells from ovaries and *Drosophila* Schneider cells (S2 cells; Fig. S6 A–B), suggesting that this localization pattern is not unique to larval brain tissues. Furthermore, we performed RNA-FISH/IF on larval brains from *Drosophila simulans*, which diverged from *D. melanogaster* 2.5 million years ago (Garrigan et al., 2012), and whose centromeres are enriched in *Jockey-3* (Chang et al., 2019). We observed centromeric foci for *Jockey-3* ORF2 at all mitotic centromeres, indicating that *Jockey-3* expression and transcripts localization is conserved in this species (Fig. S6C).

To ensure that the signal we observed with our *Jockey-3* probe sets corresponds to RNA and not DNA, we compared staining patterns between RNA and DNA-FISH protocols on brain squashes for the *Jockey-3* ORF2 probe and for a DNA-FISH OligoPaint targeting 61C7, a subtelomeric region of chromosome 3L (Chang et al., 2019). Using our RNA-FISH protocol, we could only detect the signal for *Jockey-3* produced by the ORF2 probe, while with our DNA-FISH protocol (which includes a DNA denaturation step and hybridization in the presence of an RNase cocktail) we only detected signal for the OligoPaint (Fig. S7). These experiments confirm that the *Jockey-3* signal shown in Fig. 1 corresponds to RNA and not DNA. Treatment with RNaseH (which degrades DNA/RNA hybrids) post-hybridization dramatically reduced the signal intensity of *Jockey-3* foci, indicative of degraded DNA probe/RNA hybrids. We also observed a reduction in *Jockey-3* fluorescence when we performed a pre-incubation with an RNase cocktail expected to degrade single stranded RNA prior to RNA-FISH (Fig. S8). Together, these controls indicate that the *Jockey-3* transcripts we detect at centromeres with our RNA FISH protocol are *Jockey-3* single stranded transcripts.

In addition to localizing to centromeres, *Jockey-3* transcripts also localized to non-centromeric foci on all mitotic chromosomes with the exception of chromosome 4. On average, we observed 1 non-centromeric *Jockey-3* focus per mitotic spread, with a subset of cytological regions displaying foci more frequently than others (*e.g.* middle of XL; Fig. S9). Due to gaps in our genome assembly and the limited resolution that can be obtained by microscopy, it was not possible to determine to which *Jockey-3* copies these foci correspond.

Centromeric *Jockey-3* foci were also present in interphase cells from larval brains, ovaries, and S2 cells (Fig. S10A–C). On average, larval brains interphase cells displayed <1 *Jockey-3* focus co-localizing with CENP-C, versus 2–3 non-centromeric foci (Fig. S10D). Overall, mitotic cells display approximately 3 times more *Jockey-3* foci than interphase ones (Fig. S10E). Remarkably, only 15% of interphase cells display 2 or more *Jockey-3* foci co-localizing with CENP-C versus 93% of mitotic cells (Fig. S10F). *Drosophila* centromeres are often found clustered together in interphase, which might in part account for this difference. The non-centromeric *Jockey-3* foci observed in interphase could reflect transcripts that remain associated in *cis* or unbound nuclear RNAs.

Lastly, to expand on our RNA localization studies, we designed smRNA-FISH probes for another centromeric non-LTR element, *Doc*, which is found within centromere X and 4 that shows active transcription (Fig. 1). We performed smRNA-FISH/IF on mitotic and interphase cells from larval brains squashes. Unlike *Jockey-3*, *Doc* transcripts were not detectable at the centromeres in metaphase, although the signal was visible in a few interphase cells, where it co-localized with one CENP-C focus (Fig. S11). We conclude that not all centromeric retroelements produce transcripts that localize to centromeres in metaphase.

### *Jockey-3* transcripts co-localize with their cognate sequences in *cis*

**Figure 3.**
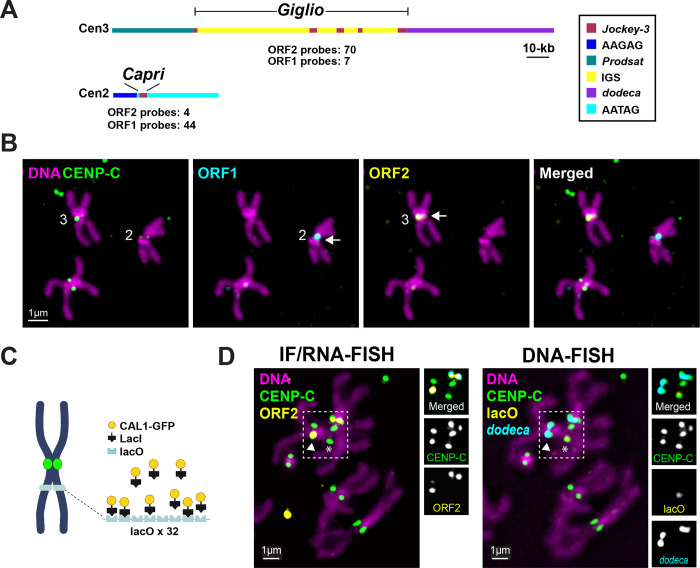
*Jockey-3* transcripts co-localize with their cognate sequences in *cis* **A** Schematic showing the organization of centromere 3 (top) and 2 (bottom) and the number of probes from the ORF1 and the ORF2 (both sense) predicted to bind to the *Jockey-3* elements therein. **B** Representative spread from RNA-FISH/IF in iso-1 flies showing the presence of *Jockey-3* signal for the ORF2 (yellow) at the centromere of chromosome 3 (arrowhead) and for the ORF1 (cyan) at the centromere of chromosome 2. CENP-C (green) and DNA stained with DAPI (magenta). Bar 1μm. **C** Schematic showing the *de novo* centromere system for chromosome 3 (lacO 80C4). Progeny containing one lacO chromosome 3, UAS-CAL1-GFP-LacI, and elav-GAL4 were analyzed by sequential IF/RNA/DNA FISH. **D** Sequential IF/RNA (leg)/DNA-FISH (right) on larval brain metaphase spreads of *de novo* centromere progeny (CAL1-GFP-LacI; lacO 80C4) showing *Jockey-3* transcripts (3’ probe; yellow) overlapping with the endogenous centromere 3 (yellow arrowhead) but not the *de novo* centromere on lacO (asterisk). CENP-C is a centromere marker (green), *dodeca* is a satellite specific for centromere 3 (cyan). The lacO array DNA FISH is shown in yellow in the right panel. Bar 1μm. N=6 brains (3 males, 3 females), n=90 cells total.

Studies in human and *Drosophila* cultured cells and in *Xenopus* egg extracts reported that different centromere and pericentromere-derived repeat transcripts can localize to centromeres either in *cis* (*i.e.* at the locus of origin; (Bobkov et al., 2018; McNulty et al., 2017)) and in *trans* (*i.e.* to all centromeres whether or not they contain complementary sequences; (Blower, 2016; Rosic et al., 2014)). Two observations from our data so far point towards *cis* localization of *Jockey-3* transcripts at the centromere. First, *Drosophila* centromere 2 contains two fragments of *Jockey-3*, one targeted by only 4 out of 44 probes in the ORF2 set and the other targeted by 44 out of 45 probes in the ORF1 set (Table 2 and Fig. 3A) and we observe robust RNA FISH signal nearly exclusively with the one targeting ORF1 (Fig. 3B and 1E). Conversely, centromere 3 *Jockey-3* copies are targeted primarily by ORF2 probes and indeed we observe strong centromeric signals for ORF2 but not ORF1. Second, the centromeric signal intensity for *Jockey-3* RNA FISH is positively correlated with the number of probes targeting that centromere (Fig. 1F and Table 1 and S1), whereas with *trans* localization, a more uniform signal intensity would be expected, irrespectively of the DNA composition of each centromere. These observations suggest that RNAs emanating from *Jockey-3* copies colocalize with their cognate DNA sequences in *cis*.

To more robustly test if *Jockey-3* transcripts can localize in *trans*, we next asked if *Jockey-3* transcripts can localize to a *de novo* centromere formed on DNA devoid of any centromere-associated repeats. We used a previously developed LacI/lacO system that efficiently forms ectopic centromeres *in vivo* via the tethering of the CENP-A assembly factor CAL1, fused to GFP-LacI, to a 10-kb lacO array inserted at the pericentromere of chromosome 3 (Palladino et al., 2020). We analyzed a total of 89 metaphase spreads from 3 male larval brains by IF with anti-CENP-C antibodies and RNA-FISH with the ORF2 probe (IF/RNA-FISH) and, After imaging, performed sequential DNA FISH to confirm the location of lacO in the same spreads. We found that, while robust localization of *Jockey-3* ORF2 transcripts at endogenous centromere 3 was clearly visible, *Jockey-3* signal was nearly never observed at the ectopic centromere on lacO (2/89; Fig. 3B–C). Together, these findings are consistent with *Jockey-3* transcripts remaining associated with the DNA sequences they originated from, similarly to what was reported for centromeric alpha-satellite transcripts in human cells (McNulty et al., 2017).

### Knockdown of *Jockey-3* RNA does not negatively affect normal centromere function

Knock-downs of alpha-satellite transcripts (McNulty and Sullivan, 2017) and of a LINE-1 element associated with a neocentromere (Chueh et al., 2009) lead to a decrease in the levels of CENP-A from the (neo)centromeres these transcripts originate from, suggesting they play a localized role in centromere maintenance or stability. In contrast, in *S. pombe,* centromere-derived transcripts are rapidly degraded by the exosome and are thus unlikely to play such a structural role, but rather appear to be byproducts of centromere transcription (Choi et al., 2011).

**Figure 4.**
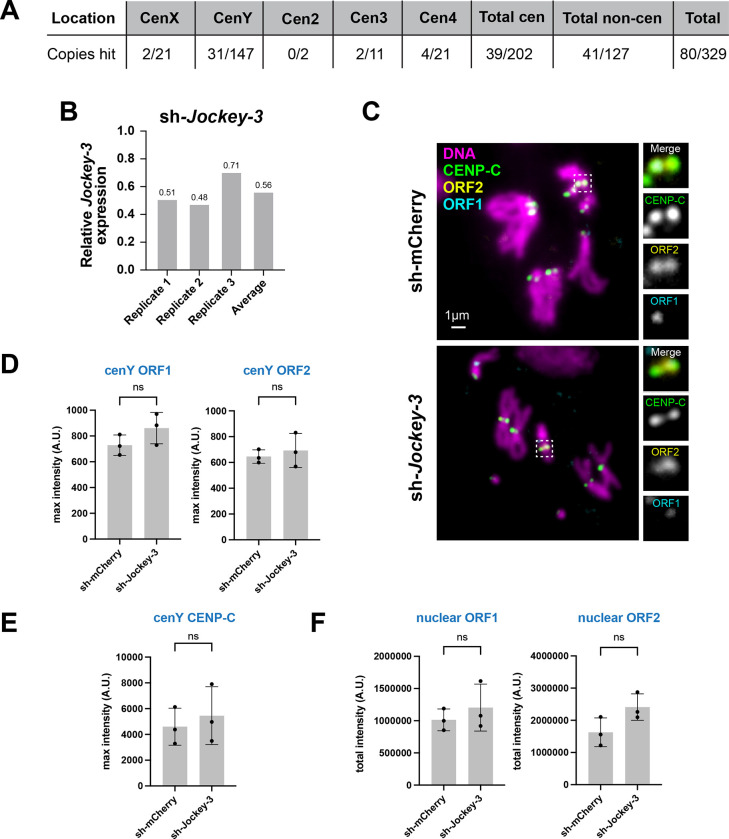
Knockdown of *Jockey-3* RNA does not negatively affect normal centromere function **A** Table showing centromeric and non-centromeric *Jockey-3* copies targeted by the sh-*Jockey-3* over the total number of *Jockey-3* copies. Targets with up to 3 mismatches are included. **B** Efficiency of *Jockey-3* knockdown determined by RT-qPCR normalized to Rp49 and set relative to sh-mcherry control in elav-GAL4 male larval brains. The average of three biological replicates are shown. The primers used here capture 72/329 (ORF2 RT primer set) *Jockey-3* copies throughout the genome. Among them, 68/72 are also targeted by the sh, 32 of which are centromeric copies (one on X, 3 on 4th, 26 on the Y, two on the 3rd). **C** Representative images of mitotic spreads from larval brains expressing sh-mcherry control and sh-*Jockey-3* stained by IF/RNA-FISH with CENP-C antibodies (green) and *Jockey-3* ORF1 (cyan) and ORF2 (yellow) probes. Insets show a zoomed image of the centromeres in the box. Bar 1μm. **D** Quantification of *Jockey-3* ORF2 and ORF2 RNA-FISH signals at the Y centromere. Bar graphs show the average fluorescence intensity for *Jockey-3* ORF2 and ORF1 at the Y centromere from sh-mcherry and sh-*Jockey-3* (unpaired t-test, p>0.05 for both the *Jockey-3* ORF2 and ORF2, N=3 brains, n=25 Y centromeres/brain). **E** Quantification of CENP-C signals at the Y centromere. The bar graph shows the average fluorescence intensity for CENP-C at the Y centromere from sh-mcherry and sh-*Jockey-3* (unpaired t-test, p>0.05, N=3 brains, n=25 Y centromeres/brain). **F** Quantification of *Jockey-3* ORF2 and ORF2 RNA-FISH signals in the total interphase cell nucleus. Bar graphs show the average fluorescence intensity for *Jockey-3* ORF2 and ORF1 in the cell nucleus from sh-mcherry and sh*Jockey-3*. (unpaired t-test, p>0.05 for both *Jockey-3* ORF2 and ORF2, N=3 brains, n=25 Y centromeres/brain).

To test the possibility that *Jockey-3* transcripts themselves play a role in centromere integrity, we designed a short-hairpin (sh) to target *Jockey-3* RNA for degradation via *in vivo* RNA interference (RNAi). As *Jockey-3* copies are heavily polymorphic in sequence and length, no single sh can target the majority centromeric or genomic copies. We therefore designed a sh targeting the RT domain in ORF2, which is present in ~27% of *Jockey-3* insertions in the genome, targeting as many centromeric and non-centromeric copies as possible (Fig. 4A), and generated transgenic flies expressing the sh-*Jockey-3* under the GAL4 UAS promoter.

To verify the effectiveness of the knock-down, we induced sh-*Jockey-3* expression under the neural elav-GAL4 driver, isolated total RNA from larval brains, and measured *Jockey-3* expression by RT-qPCR, using primers outside of the sh-*Jockey-3* target. These primers capture 72/80 *Jockey-3* copies targeted by the short hairpin, including 2 centromeric copies on the X, 27 on the Y, 2 on 3, and 3 on 4, all of which copies were confirmed as being active by PRO-seq. Across three biological replicates, we found that sh-*Jockey-3* expression was reduced by ~50% in sh-*Jockey-3* compared to a sh-mcherry control (Fig. 4B). However, measurements of the RNA-FISH signal intensity showed no significant change for *Jockey-3* ORF1 or ORF2 at the Y centromere (Fig. 4C–D). Similarly, we did not observe a decrease in CENP-C intensity at the Y chromosome (Fig. 4E), which would have been indicative of a centromere assembly defect, nor did we detect an increase in aneuploidy (N=3 brains, n=25 spreads each, 1.33% aneuploid in sh-*Jockey-3* versus 6.7% in control, p=0.2).

RNAi based knockdowns typically affect transcripts post-transcriptionally and their effectiveness in knocking down nuclear RNAs is unclear (discussed in (Bassett et al., 2014)). To determine if the nuclear pool of *Jockey-3* transcripts is reduced upon RNAi, we quantified the total nuclear fluorescence intensity of *Jockey-3* and found no significant change compared to the control (Fig. 4F), suggesting that the decrease in expression observed by RT-qPCR (Fig. 4B) reflected changes in the cytoplasmic pool of *Jockey-3*. Consistent with the lack of mitotic defects, expression of the hairpin under the eyeless-GAL4 driver in adult eyes did not cause any disruptions to eye morphology compared to the control (data not shown). We also observed similar progeny viability and fertility in flies expressing sh-*Jockey-3* compared to controls (data not shown). These findings suggest that the cytoplasmic pool of *Jockey-3* RNA is not important for centromere integrity, chromosome segregation, or viability. However, given that this approach did not successfully deplete the nuclear pool of *Jockey-3*, we cannot rule out that nascent *Jockey-3* RNA may play a role as a *cis*-acting ncRNA at centromeres.

### CENP-A chromatin profiling reveals a link between nascent transcription and CENP-A association for full-length *Jockey-3* copies in the genome

**Figure 5.**
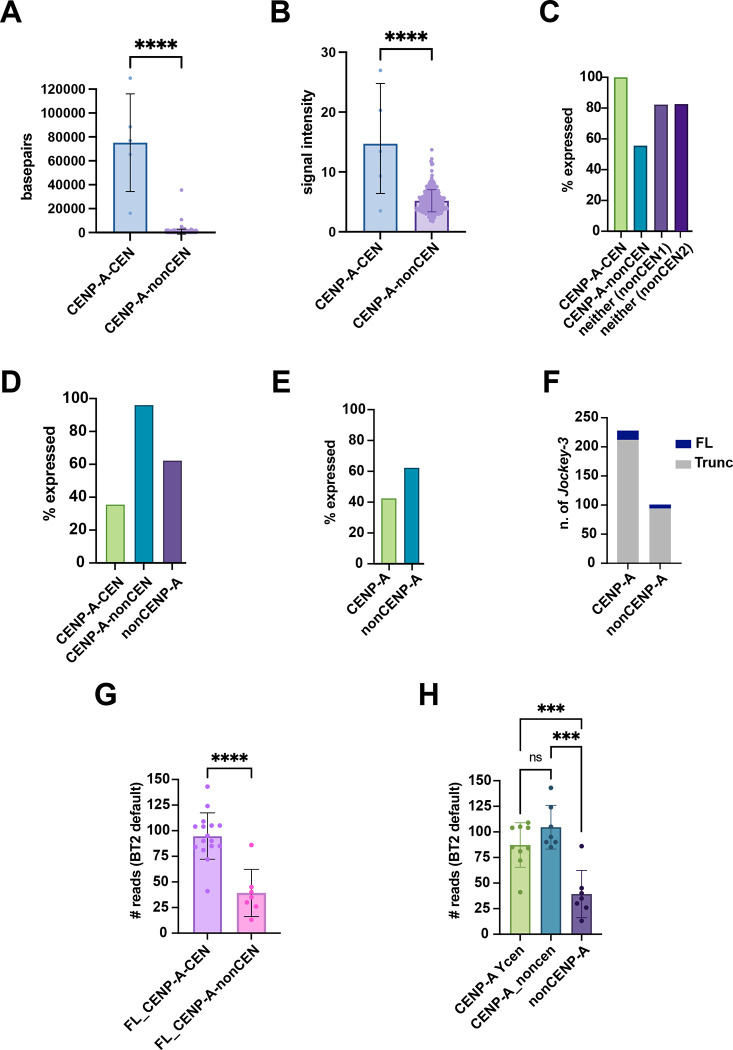
Relationship between CENP-A occupancy and transcription at centromeres and other genomic locations **A** Scatter boxplot showing CENP-A domain size (in base pairs) between centromeric (n=5) and non-centromeric (n=333) loci based on MACS2 peak calls from CUT&Tag data. An unpaired t test determined a statistically significant difference (****; p < 0.0001; Student’s t-test). Standard deviation error bars are shown. **B** Scatter boxplot showing CENP-A peak signal intensity between centromeric and non-centromeric loci based on MACS2 peak calls from CUT&Tag data. Signal intensity was averaged across each CENP-A domain. An unpaired t test determined a statistically significant difference (****; p < 0.0001; Student’s t-test). Standard deviation error bars are shown. **C** Bar graph illustrating the proportion of CENP-A domains expressed within and outside the centromere. Additionally, a non-CENP-A associated control was included for comparison. PRO-seq mapping was done with Bowtie 2 default “best match” using paired-end reads. Expression is defined as having either at least 2 PRO-seq read overlaps. **D** Bar graph illustrating the proportion of *Jockey-3* copies expressed per group, where groups are based on CENPA and centromeric association. PRO-seq mapping was done with Bowtie 2 default “best match” using paired-end reads. Expression is defined as having at least two PRO-seq read overlaps. **E** Same as shown in Fig. 6D, except *Jockey-3* copies found within CENP-A domains (regardless of centromeric association) are combined into one group (“CENP-A”). **F** Distribution of *Jockey-3* copies as a stacked bar graph. Copies are grouped by whether they are found within CENP-A domains (centromeric and non-centromeric) or outside CENP-A domains, as well as their status as a full-length (blue) or truncated element (gray). **G** PRO-seq read density scatter boxplot of full-length *Jockey-3* copies comparing those found within CENP-A domains (centromeric and non-centromeric) and those found outside CENP-A domains. Mapping was done with Bowtie 2 default “best match” using paired-end reads. An unpaired Student’s t-test determined a statistically significant difference (****; p < 0.0001). Standard deviation error bars are shown. **H** Same as shown in Fig. 5G, except *Jockey-3* copies found within CENP-A domains are split by centromeric (only found on chromosome Y) or non-centromeric location. Unpaired t tests (Student’s t-test) were performed between each group (***, p < 0.001; ns (non-significant), p > 0.05).

*Jockey-3* is the most enriched repeat in CENP-A chromatin immunoprecipitations (Chang et al., 2019), but this retroelement is also present elsewhere in the genome (Table S1). However, the association between CENP-A and these non-centromeric copies has not been explored. Furthermore, we do not know if the association with CENP-A and the transcriptional activity of *Jockey-3* or are interconnected. To investigate these questions, we used CUT&Tag (Kaya-Okur et al., 2019) from 0–12h embryos, mapped the resulting sequencing data to the heterochromatin-enriched genome assembly from (Chang et al., 2019) and identified significant peaks. We identified five centromeric CENP-A domains (Fig. S12) and 333 non-centromeric domains (Table 3; Table S4). The non-centromeric CENP-A domains were smaller on average and had lower CENP-A signal intensity than the centromeric domains (Fig. 5A–B). Next, we asked if CENP-A occupancy correlates with nascent transcription. We identified CENP-A domains with at least two PRO-seq reads overlap and found that 100% of centromeric CENP-A domains show nascent transcription, while 56% of non-centromeric CENP-A domains are transcribed. Two random intervals of non-centromeric CENP-A domain size also showed transcription (Fig. 5C, Table S4). Thus, CENP-A chromatin is transcribed, especially at centromeres.

We then examined whether the transcription of *Jockey-3* insertions correlated CENP-A occupancy. There are 26 copies of *Jockey-3* that fall within a non-centromeric CENP-A domain, 202 that fall within a centromeric CENP-A domain, and 101 that fall in neither (Table S6). We found that, while 36% of *Jockey-3* copies within the centromeric CENP-A domains are expressed, this percentage increases for *Jockey-3* copies at non-centromeric CENP-A regions, with 96% of *Jockey-3* copies showing expression. Expression of *Jockey-3* copies not CENP-A associated is also high at around 60% (Fig. 5D; Table S1 and Table S5). However, when we compare all CENP-A associated *Jockey-3* copies versus all non-CENP-A associated ones, the difference in the percentage of active *Jockey-3* elements is only 43% versus 62%, respectively (Fig. 5E; Table S1 and Table S5). We conclude that although there is an enrichment of *Jockey-3* elements associated with CENP-A versus not (228/329, or 69%; Fig. 5F and Table S6), the expression of *Jockey-3* in embryos appears to be independent of its association with CENP-A. However, when we consider only full-length *Jockey-3* copies, which are the most highly expressed copies in the genome (Fig. 1B), we see a strong and positive correlation between association with CENP-A and active transcription (Fig. 5G; Table S1), regardless of centromeric location. Breaking down the data by where these full-length *Jockey-3* copies are located, it is clear that CENP-A association, rather than centromeric location, is driving this transcription (Fig. 5H). From these analyses, we conclude that full-length *Jockey-3* copies are more highly expressed when coupled with CENP-A chromatin. Along with our results showing that full-length *Jockey-3* copies are the source of most of *Jockey-3* transcripts (Fig. 1B) and that the Y centromere displays the strongest RNA-FISH signal amongst all centromeres (Fig. 2), we conclude that much of the full-length *Jockey-3* transcription can be attributed to its association with CENP-A.

It is noteworthy to mention that both PRO-seq and CUT&Tag were performed on nuclei from embryos, thus reflecting the transcriptional and chromatin profiles of primarily interphase cells. In contrast, our RNA-FISH experiments showing higher frequency of detection of *Jockey-3* at centromeres compared to non-centromeric locations were performed on metaphase chromosomes (Fig. S10C). We infer that the proportion of *Jockey-3* transcripts emanating from centromeres versus non-centromeric regions is likely to be higher in mitotic cells.

### *lacO* transcription is coupled with *de novo* centromere formation

Having shown that centromeric *Jockey-3* copies are expressed, and that expression of full-length copies is strongly correlated with CENP-A occupancy, we next investigated if *de novo* centromeres are also transcribed. *De novo* centromeres have been shown to form efficiently both *in vitro*, in *Drosophila* S2 cells (Chen et al., 2015), and *in* vivo, in somatic *Drosophila* tissues (Palladino et al., 2020), when the CENP-A chaperone CAL1 fused to GFP-LacI is tethered to a lacO array inserted within the genome. Upon its tethering to the lacO array in S2 cells, CAL1, alongside the elongation factor FACT and RNA polymerase II, initiate transcription of non-endogenous sequences belonging to the inserted lacO array (Chen et al., 2015).

**Figure 6.**
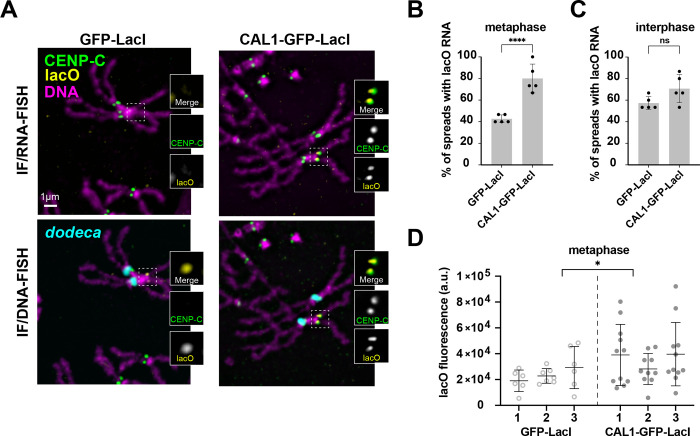
Transcript accumulation at a *de novo* centromere **A** Sequential IF/RNA/DNA FISH on larval brains from GFP-LacI and CAL1-GFP-LacI/3^peri^, both (lacO array at 80C4). IF for CENP-C is shown in green. RNA and DNA FISH with a lacO LNA probe are shown in yellow. DNA FISH for *dodeca* is shown in cyan. **B** Bar graphs showing the frequency of lacO transcription in GFP-LacI and CAL1-GFP-LacI/3^peri^ in metaphase and **C** in interphase (Fisher’s exact test, N = 5 brains, n = 15 spreads/brain). **D** Scatter plot showing the fluorescence intensity of lacO RNA-FISH in GFP-LacI and CAL1-GFP-LacI/3^peri^ in metaphase (nested t-test, N = 3 brains, n = 6–11 spreads/brain).

To determine if the DNA associated with a *de novo* centromere becomes transcribed, we used an oligo lacO probe to detect lacO-derived transcripts by RNA-FISH in larval progeny expressing CAL1-GFP-LacI or a GFP-LacI control under the neural elav-GAL4 promoter and heterozygote for a pericentric 10-kb lacO array inserted at 3L (3^peri^ at 80C4; (Palladino et al., 2020)). As expected from previous studies, expression of CAL1-GFP-LacI results in ectopic centromere formation at the 3^peri^ lacO array in more than 80% of spreads (Palladino et al., 2020). We performed sequential IF-RNA/DNA-FISH on mitotic spreads from larval brains in elav-GAL4 CAL1-GFP-LacI and GFP-LacI/lacO expressing progeny. IF for CENP-C was used to identify active centromeres and lacO RNA-FISH allowed us to establish if transcripts are visible at ectopic centromeres. After imaging metaphase spreads, we processed the slides for DNA-FISH with the same lacO probe to identify the position of the lacO array alongside a probe for the peri/centromeric satellite *dodeca* to identify the endogenous centromere 3s and re-imaged the same spreads. We found that CAL1-GFP-LacI/3^peri^ spreads display lacO RNA-FISH signal with a significantly higher frequency compared to the GFP-LacI control (Figure 6A–B). We also found that there is no significant difference in lacO transcription frequency between CAL1-GFP-LacI/3^peri^ and GFP-LacI/3^peri^ in interphase cells (Fig. 6C), suggesting that the higher transcription frequency observed in CAL1-GFP-LacI/3^peri^ spreads is specific to metaphase. To determine if lacO expression levels are different between GFP-LacI and CAL1-GFP-LacI/3^peri^ spreads, we measured lacO RNA fluorescence intensity across three brains for both genotypes and found that CAL1-GFP-LacI/3^peri^ shows higher lacO RNA signal intensity than GFP-LacI/3^peri^ in the GFP-LacI/3^peri^ control (Fig. 6C). Collectively, these experiments show that although lacO is transcribed in the absence of an ectopic centromere, transcription is observed at a higher frequency and at higher levels when an ectopic centromere is present, suggesting that the formation of a *de novo* centromere stimulates local transcription. These results are consistent with previous reports in human neocentromeres (Chueh et al., 2009; Murillo-Pineda and Jansen, 2020; Naughton et al., 2022) and *de novo* centromeres in S2 cells (Chen et al., 2015) showing increased transcription upon CENP-A chromatin formation at non-centromeric sites. These results further underscore the correlation between CENP-A deposition in mitosis and an increase in transcription.

## Discussion

In this study, we examined the transcriptional landscape of *Drosophila* centromeres and identified widespread transcription across these regions. Building upon the recent assembly and annotation of *Drosophila* centromeres, we found that the centromere-enriched retroelement *Jockey-3* (Chang et al., 2019), produces transcripts that accumulate at all centromeres, a localization that is conserved in *D. simulans*. *Jockey-3* transcripts remain associated with their cognate DNA sequences and do not diffuse to other native nor *de novo* centromeres. The enrichment of these transcripts at centromeres is especially evident in metaphase, a time that coincides or precedes (depending on cell types and species) metazoan CENP-A deposition (Dunleavy et al., 2012; Jansen et al., 2007; Lidsky et al., 2013; Mellone et al., 2011; Pauleau et al., 2019; Schuh et al., 2007). A boost in transcription before or around the time of deposition could prime chromatin by removing placeholder histone H3 (Dunleavy et al., 2011) to allow the assembly of CENP-A nucleosomes. Consistent with this model, active RNA polymerase II (RNAPII) and/or transcriptional activity has been reported at metaphase centromeres in *Drosophila* (Bobkov et al., 2018; Rosic et al., 2014) and human cell lines (Chan et al., 2012; Hoyt et al., 2022; McNulty et al., 2017).

An alternative explanation for why RNAPII at centromeres is highest at metaphase may be because it is lost from arms upon cohesin degradation in prophase, yet persists in metaphase at centromeres in human cells (Perea-Resa et al., 2020). However, this phenomenon does not preclude the resulting transcription from providing the beneficial effect of freeing up chromatin for CENP-A deposition. The use of transcriptional inhibitors for a short window of time has the potential to inform on whether the act of transcription is important for CENP-A maintenance. In *Drosophila* S2 cells, transcriptional inhibition stabilized the chromatin association of new CENP-A at centromeres (Bobkov et al., 2018). In contrast, another study used RNA polymerase inhibitors injected into *Drosophila* embryos and quantified GFP-CENP-A intensity across two cell divisions. The authors did not detect a decrease in GFP-CENP-A signal, which would be expected if transcription was required for *de novo* GFP-CENP-A deposition (Ghosh and Lehner, 2022). However, it is unclear if CENP-A deposition during the rapid divisions of early embryos involves eviction of place-holder histone H3. As these inhibitors affect global transcription indiscriminately, centromere-specific inhibition of transcription is needed to resolve whether it is required for CENP-A maintenance.

There are 329 copies of *Jockey-3*, 202 of which (61%) are found within the five centromere contigs (Chang et al., 2019; Hemmer, 2023). Analyses of nascent transcripts reveal that the *Jockey-3* copies present within the centromeres are not expressed at higher levels than elsewhere in the genome –in fact, at least in interphase, *Jockey-3* elements within the centromeres are overall expressed at lower levels– suggesting that *Jockey-3* elements are active irrespective of their genomic location. Indeed, the accumulation of these repeats at the centromere might underscore natural selection for transcriptionally active elements in these regions. These results are consistent with studies in human RPE cells that showed alpha-satellite transcripts are produced from arrays outside of the active human centromere region (McNulty et al., 2017).

Full-length *Jockey-3* copies contribute the most to overall *Jockey-3* transcription, and most of these full-length copies are found at non-centromeric loci (14/23). Interestingly, we find that the expression of these full-length *Jockey-3* copies is strongly positively correlated with CENP-A occupancy. Our PRO-seq profiles reflect nascent transcription in interphase, and in these cells the co-localization of *Jockey-3* RNA signal with centromeres occurs less frequently and at fewer centromeres than in metaphase. In contrast, RNA-FISH experiments on metaphase chromosomes reveal bright *Jockey-3* RNA foci primarily at centromeres. The observation that transcripts from the expressed centromere-associated retroelement *Doc* do not localize to metaphase centromeres, unlike those from *Jockey-3*, implies that *Jockey-3* has a unique capability for enhanced transcription during this stage.

Our finding that *de novo* centromeres are coupled with transcriptional activation of the underlying DNA specifically in metaphase reinforces the model that CENP-A deposition and transcription go hand in hand. However, we cannot distinguish between transcriptional activation of lacO being caused by CAL1 tethering, given that CAL1 is known to interact with RNAPII and FACT (Chen et al., 2015), or being linked to active CENP-A deposition. The latter possibility would be consistent with recent studies in human neocentromeres showing that neocentromere formation is associated with transcriptional activation and increased chromatin accessibility (Murillo-Pineda et al., 2021; Naughton et al., 2022).

While centromere-associated *Jockey-3* transcripts are visible with high frequency in metaphase, non-centromeric foci are more rare and certainly fewer than the 127 known non-centromeric *Jockey-3* insertions, or the 14 full-length non-centromeric copies. In interphase too, the number of non-centromeric foci is much smaller than the number of *Jockey-3* copies. It is possible that different insertions alternate between active and inactive states, with more activity occurring in interphase. However, it is more likely that only a subset of full-length *Jockey-3* copies produce nascent transcripts detectable by RNA-FISH at any given time.

*Jockey-3* is enriched at the centromere compared to the rest of the genome (Chang et al., 2019) and is a recently active retroelement with weak insertional bias for the centromere in population studies (Hemmer et al., 2023). The centromeres of three species within the *Drosophila simulans* clade, *D. simulans*, *D. mauritiana*, and *D. sechellia*, and *D. melanogaster* display a remarkable turnover in sequence composition suggesting the existence of a genetic conflict between satellites and retroelements (Courret et al., 2023). To ensure their own propagation through generations, these selfish genetic elements appear to compete for dominance at the centromere, a region with low recombination that can tolerate variation in sequence composition without loss of functionality. Since *Jockey-3* is targeted by piRNA-mediated silencing in the germline (Hemmer et al., 2023), its preferential insertion at centromeres could provide an advantage for its continuous propagation since centromeres are typically not associated with heterochromatin (Courret et al., 2023; Hemmer et al., 2023; McKinley and Cheeseman, 2016; Sullivan and Karpen, 2004). In turn, *Jockey-3* could benefit the host by promoting local transcription, which would facilitate chromatin remodeling during CENP-A deposition. Changes in expression for LINE1 modulate global chromatin accessibility during early mouse embryo development independently of both the LINE1 RNA or its protein products (Jachowicz et al., 2017). Similarly, *Jockey-3* expression could promote local chromatin accessibility at centromeres.

Global analyses of the chromatin-associated non-coding transcriptome in human embryonic stem cells showed that most RNA-DNA interactions are proximity based, with virtually none occurring in *trans*. Furthermore, TE-derived RNAs are frequently found associated with chromatin (Limouse et al., 2023). Our results showing *cis* localization of *Jockey-3* are consistent with these findings. Even though we did not observe RNA-FISH signal in metaphase for the centromere-associated *Doc* retroelement, it is possible that additional centromere-derived RNAs contribute to the overall regulatory output of RNA-chromatin interactions at the centromere, similar to that proposed for genes (Limouse et al., 2023). Alternatively, *Jockey-3* RNAs may simply be an incidental byproduct of the element’s transcription with no further regulatory role (Bassett et al., 2014). To investigate this, more targeted tools will be needed to specifically inhibit transcription of centromeric repeats and assess local effects on CENP-A chromatin. Moreover, RNA localization evidence does not differentiate between RNAs that are tethered to the centromere through active transcriptional machinery from RNAs complexed with centromeric proteins. Future work will need to explore if the retention, the metaphase transcription of *Jockey-3*, or neither, require the centromere environment to take place.

*Jockey-3* transcripts form distinct, bright foci at metaphase centromeres, bearing similarity to RNA-rich nuclear condensates like histone clusters, nucleoli, and Cajal bodies (Decker et al., 2022). RNA has the capacity to initiate condensate formation, supporting the nucleation of additional RNAs and proteins (Rhine et al., 2020). In *S. pombe*, clustering of the centromeres by the Spindle Pole Body facilitates CENP-A assembly through this structure’s ability to attract high concentrations of CENP-A and its assembly factor (Wu et al., 2022). It is possible that high concentrations of *Jockey-3* transcripts produced in metaphase may aid in the maintenance of centromeres by attracting elevated levels of *Drosophila* CENP-A and its assembly factor CAL1 (Chen et al., 2014). This mechanism could depend more on the origin of the RNA (specifically, its derivation from centromeres) rather than its unique sequence.

## Methods

### *Drosophila* stocks and handling

Flies were reared on standard cornmeal, molasses, and yeast food (https://bdsc.indiana.edu) at 25°C, except for crosses for RNAi and sh-mediated knockdowns, which were carried out at 29°C. Experiments were performed in the following *D. melanogaster* stocks: laboratory stock iso-1 (Bloomington Drosophila Stock Center stock no. 2057: y1; Gr22b^iso−1^ Gr22d^iso−1^ cn1 CG33964^iso−1^ bw1 sp1; MstProx^iso−1^ GstD5^iso−1^ Rh61); lacO ^(3peri^, 80C4); UAS-CAL1-GFP-LacI and UAS-GFP-LacI maintained as heterozygous lines with the T(2;3)TSTL double balancer (Chang et al., 2019); sh-mCherry (Bloomington Drosophila Stock Center stock no. 35785) and sh-*Jockey-3*. The GAL4 driver used was elav-GAL4 balanced with T(2;3)TSTL double balancer. The *D. simulans* stock used is w501 (gig of Andy Clark).

For all knockdowns, elav-GAL4 balanced with T(2;3)TSTL males were crossed with sh-virgin females at 29°C. Non-tubby larvae, which carried both elav-GAL4 and sh, were selected for dissections.

The sh-*Jockey-3* line was generated by PhiC31-mediated integration of pVALIUM20-sh-*Jockey3* at the abP2 landing site After injection by a commercial service (Best Gene). The *Jockey-3* hairpin was designed against the reverse-transcriptase region of *Jockey-3* using the DSIR website (http://biodev.extra.cea.fr/DSIR/DSIR.html), picking the one with the highest score. The sequences targeting *Jockey-3* were: 5’-ACGCTGGAACATCATGATCAA (Passenger strand) and 5’-TTGATCATGATGTTCCAGCGT (Guide strand). The oligos ordered included the passenger and guide strands flanked by standard flanking sequences. The resulting oligos were: 5’-ctagcagtACGCTGGAACATCATGATCAAtagbatabcaagcataTTGATCATGATGTTCCAGCGTgcg (Top strand) and 5’aabcgcACGCTGGAACATCATGATCAAtatgcbgaatataactaACGCTGGAACATCATGATCAAactg (Bottom Strand). These top and bottom strands were annealed together creating overhangs and ligated into pVALIUM linearized with NheI and EcoRI.

### Cell culture

*Drosophila* Schneider (S2) cells were grown in Schneider’s media containing 10% FCS and anti-biotic/anti-mycotic mix at 25°C. Cells were passaged twice a week by diluting a cell resuspension to a million cells/ml.

### Stellaris probe design

Custom probes were designed using the Stellaris FISH probe designer. Probes were designed against the *Jockey-3* consensus sequence using ORF1 and ORF2 as targets. See Table of reagents for probes sequences.

### RNA extraction from brains and RT-qPCR

20–30 male larval brains were dissected in ice cold PBS DEPC and preserved in 150μl RNA later at −20°C. PBS DEPC was added to the brain suspension and spun to pellet the brains. The PBS/RNA later was removed and the brains were lysed in 300μl of TRIzol using a motorized pestle. RNA was extracted with Zymo Direct-zol RNA MiniPrep Kit (Cat#: 11–330) according to manufacturer’s instructions, except the in-column DNase I treatment was repeated twice. Samples were then treated with Turbo DNAse 2 to 3 times and then purified with the RNA Clean and Concentrator-5 Kit (Zymo Research Cat#: 11–325) according to the manufacturer’s instructions. cDNA was prepared with iScript Reverse Transcription Supermix following the manufacturer’s instructions. PCR was used to check cDNA quality and no DNA contamination in the no reverse transcriptase samples. qPCR was performed with iTaq Universal SYBR Green Supermix in 96 well plates, and ran on a BioRad qPCR thermocycler. Relative quantity was calculated with the Pfaffl method (Pfaffl, 2001).

The PCR cycle was as follows: 95 °C 3 min for initial denaturation, then followed by 40 qPCR cycles. Each cycle has denaturation at 95 °C for 10s, annealing at 55°C for 20s and extension at 72°C for 20s.

### Primer design for targeting *Jockey-3*

Primers targeting *Jockey-3* copies with centromere contigs (Chang et al., 2019) were designed using the primer design tool in Geneious Prime and checked for off-target hits against our genome assembly from (Chang et al., 2019) using Geneious Prime Specificity testing tool,Primers that targeted *Jockey-3* copies that pro-seq reads map to were selected. Primers were validated using cDNA and confirmed the presence of a single band using gel electrophoresis.

Primers targeting multiple *Jockey-3* copies were designed against ORF2 from the *Jockey-3* consensus sequence using the Primer Design toolkit in Geneious Prime.

### Metaphase spread preparations from larval brains

All solutions were made up in DEPC milliQ water. Third instar larval brains were dissected (2–3 brains/slide) in PBS and all attached tissue and mouth parts were removed with forceps. Brains were immersed in 0.5% sodium citrate solution for 8 min in a spot well dish then moved to a 6μl drop of 45% acetic acid, 2% Formaldehyde on a siliconized (Rain X-treated) coverslip for 6 min. A poly-lysine coated glass slide was inverted and placed on the brains to make a sandwich. After flipping the slide and gently removing excess fixative between bibulous paper, the brains were squashed with the thumb by firmly pressing down. Slides were then immersed in liquid nitrogen and the coverslip was flipped off using a razor blade. Slides were then transferred to PBS for 5 min to rehydrate before proceeding with RNA-FISH/IF or IF/RNA-FISH. Monolayers brain preparation were performed using the same procedure except that acetic acid was omitted from the fixative.

### Mitotic spread preparations from S2 cells

3×10^5^ Schneider (S2) cells were collected in a tube for each slide and media was added to reach a volume of 475μl. The cells were treated for 1 hr with 0.5μg/ml colcemid (Sigma Aldrich) to induce mitotic arrest. Cells were then spun at 600g for 5 min in a centrifuge and resuspended in 250μl of 0.5% sodium citrate (DEPC treated) for 8 min. The cell suspension was loaded into a cytofunnel and spun for 5 min at 1200 rpm onto a poly-lysine coated slide using a cytocentrifuge (Shandon Cytospin 4, Thermo Fisher Scientific). The slides were immediately transferred to a coplin jar containing 100 ml of fixative (45% acetic acid and 2% formaldehyde in DEPC water) for 6 min. Slides were then washed 3 times with PBST (0.1% Triton) for 5 min while rocking at room temperature. Slides were stored in 70% ethanol at 4°C until IF/RNA-FISH.

### Mitotic spread preparations from ovaries

Ovary mitotic preparations were conducted as in (Hanlon et al., 2018). Mated adult females were anesthetized with CO_2_, then moved to a fresh 50 μL drop of PBS. Whole ovaries were dissected out and the carcass discarded. Using a needle, the tips of the ovaries were separated from later stages and immersed in 0.5% sodium citrate for 5 min, followed by fixation for 4 mins in 2 mL of fixative solution (45% acetic acid, 2.5% formaldehyde). Fixed tissues were moved to a 3 μL drop of 45% acetic acid on a siliconized coverslip (Rain X) and gently teased apart with a needle. A poly-L lysine coated glass slide was inverted onto the coverslip and pressed gently to spread the liquid to the edges of the coverslip. The slide and coverslip were squashed for 2 minutes using a hand clamp (Pony Jorgensen 32225), then immersed into liquid nitrogen for at least 5 minutes. Coverslips were immediately removed using a razor blade. The slide was then dehydrated by placing it in ice cold 70% ethanol for 2 hr at 4°C, and processed for RNA-FISH/IF.

### RNA-FISH/IF

Slides were immersed in PBST (0.1% Triton) and rocked for 10 min 3 times. Slides were transferred to 70% ethanol at 4°C overnight. Slides were rehydrated in PBST for 5 min and washed in wash buffer (2x SSC and 10% formamide) for 5 min while rocking. Without drying the brains, 50μl probe mix containing 45μl of Hybridization buffer (Stellaris), 5μl Formamide (10% formamide final) with 0.5μl of Stellaris smRNA FISH probes (Stellaris *Jockey-3* ORF1, ORF2, ORF2 antisense, *Doc*, *Rox1*, 1:100). Brains were covered with a HybriSlip coverslip, sealed with rubber cement to prevent evaporation, and incubated at 37oC overnight in a humid chamber. Slides were then rinsed twice with wash buffer, washed twice in washing buffer for 30 min, and three times with 2X SSC for 10 min while gently shaking at RT. Slides were then post-fixed for 10 min in the dark in 100μl of 3.7% formaldehyde in PBS DEPC.

After 3 additional 5 min washes in PBST, the slides were then transferred to a coplin jar containing blocking buffer (1% BSA in PBST; PBS, 0.1% Triton-X ) for 30 min while rocking. 50 μl of primary antibodies (anti-CENP-C guinea pig polyclonal antibodies, 1:500) diluted in blocking buffer were applied to the slides, covered with parafilm and stored in a dark chamber at 4°C overnight. The following day, slides were washed 4 times with PBST for 5 min while rocking. Secondary antibodies (goat anti-guinea pig A488, 1:500) diluted in blocking buffer were applied to the brains, covered with a square of parafilm and incubated at room temperature for 1 hr. Slides were then washed 4 times in PBST for 5 min while rotating and again quickly in PBS for 3 min. Slides were mounted using SlowFade Gold containing 1μl/ml DAPI and a 22×22mm coverslip sealed with nail polish. The slides were stored in a dark environment to dry for 10 min before imaging.

### IF/RNA-FISH

Slides containing squashed larval brains were washed 3 times with PBST for 5 min on a rotator and transferred to 70% ethanol diluted at 4°C for 1 hr. Slides were then rehydrated for 5 min in PBST and processed for IF as described in the RNA-FISH/IF method above. After washing off the secondary antibodies, the slides were then processed for RNA-FISH without post-fixing, using Stellaris probes for *Jockey-3* and a lacO LNA probe Slides were mounted as described for RNA-FISH/IF.

### Sequential IF/RNA-FISH/DNA-FISH to detect lacO RNA at *de novo* centromeres

IF/RNA-FISH samples (anti-CENP-C guinea pig 1:500; lacO LNA, *Jockey-3* ORF2) were imaged and the list of points visited was saved. Coverslips were removed with a razor blade and the slides were washed in PBS for 10 min at room temperature while rocking. Slides were then washed three times with 4X SSC for 3 min, once with 2X SSCT for 5 min, and once with 50% formamide 2X SSC for 5 min at room temperature while rocking. 50 μl probe mix containing 13.5 μl 4X hybrid mix (8X SSC, 0.4% Tween20, 40% dextran sulfate, 34 μl formamide, 2μl RNase cocktail, 0.5 μl lacO LNA probe (1:100), 0.5 μl *dodeca* LNA probe (1:100) were added to the slide, covered with a hybrislip and sealed with rubber cement. Slides were incubated at 95°C for 5 min in a slide thermal cycler (Epperndorf) then transferred to a humid chamber and incubated at 37 °C overnight in the dark. After incubation, the hybrislip and rubber cement were removed. Slides were then washed once at 37°C with 0.1X SSC for 10 min and twice at room temperature with 0.1X SSC for 10 min while rocking. Slowfade Gold containing DAPI was applied to the brains, covered with 22X40 mm or 22X22 mm coverslips, and sealed with nail polish. Imaging was performed by re-visiting the same point list.

### RNAse treatments and quantification

For the RNAseH treatments, male 3rd instar larval brain monolayers from a line expressing CID-EGFP under the control of the CID regulatory sequences were processed for RNA-FISH using the *Jockey-3* ORF2 probe. Two slides were prepared. The following day, samples were imaged and point locations were recorded. Following imaging of these two pre-treatment slides, the coverslips were removed and the slides were briefly rinsed in PBS. RNAseH treatment was performed with 10U of RNAseH (cleaves the RNA when coupled with DNA; NEB) incubated for 2hr at 37°C in a dark humid chamber on one the slides, while the control slide was treated in the same way omitting the RNAseH but including the buffer diluted in water. Slides were then washed once with PBS and mounted as described. The slides were then reimaged using the same settings as before, with the same points revisited. Quantification of the samples were done by counting the number foci of eCID-GFP and *Jockey-3* ORF1 probes within cells between the pretreatment and post-treatment. Values were plotted using Prism as a scatter plot. Statistical analysis was conducted using the t-test (unpaired).

For the RNAse cocktail treatment, we generated male 3rd instar larval brain monolayers from eCID-GFP lines. Prior to RNA-FISH probe hybridization, 8X RNAse cocktail (RNAseA and RNAseT1, both targeting single-stranded RNA; Thermo Fisher) diluted in PBS was added to one slide (treated), while the other slide (untreated) only contained PBS. Samples were incubated at 37°C for 30 min. Samples were then washed for 5 min in PBS and hybridized with the *Jockey-3* ORF2 probe and Rox1 probes RNA-FISH. The following day the samples were imaged and point locations were recorded. Quantification of the samples was done by counting the number eCID-GFP and *Jockey-3* ORF2 foci within cells (N=100 cells) for both samples. Values were plotted as a scatter plot using Prism. Statistical analysis was conducted using the t-test (unpaired).

Our attempts to degrade the *Jockey-3* RNA-FISH signal from metaphase spreads with RNAseH and RNAse cocktail treatments was not successful, despite seeing *Rox1* signal become very weak or disappear. We hypothesize that the centromere/kinetochore protects *Jockey-3* RNA from degradation. We also performed these treatments After reversing the crosslinking at 80°C for 8 min as described in (Bobkov et al., 2018). However, heat treatment eliminated all *Jockey-3* RNA-FISH signal even in the absence of any RNAse, precluding us from drawing any conclusions from these experiments.

### Imaging

All images were acquired at 25°C using an Inverted Deltavision ULTRA (Leica) equipped with a sCMOS pco.edge detector camera and with either a 100x/1.40 NA or 60x/1.42 NA oil objective using 0.2μm z-stacks. Mitotic spreads were imaged using the 100x objective. Tissue monolayers were imaged using either the 60x/1.42 NA or 100x/1.40 NA oil objectives. Image acquisition was performed using DeltaVision Ultra Image Acquisition software and image processing was performed using softWoRx software (Applied Precision). Images were deconvolved for 5 cycles using the conservative setting. All stellaris probes for RNA-FISH were excited for 0.5s at 100% transmission for each z-slice image. Following deconvolution, images were quick-projected as maximum intensity projections using in-focus z-slices, a uniform scale was applied before saving images as Photoshop files. Images were minimally adjusted using Photoshop (Adobe) and assembled into figures in Illustrator (Adobe).

### Colocalization quantification for *Jockey-3* at centromeres

Metaphases were inspected in the CENP-C channel to identify centromeres and the presence of *Jockey-3* signal was determined by eye and recorded as colocalizing if present in at least one sister.

### Colocalization quantification for *Jockey-3* at *de novo* lacO centromeres

The presence of dicentrics causes chromosome breaks and rearrangements, making the identification of chromosomes difficult. Therefore, we selected metaphases with intact chromosome 3’s (identified with *dodeca* DNA-FISH) and with CENP-C signal at the 3peri location (identified with lacO DNA-FISH) for quantification. For the cis/trans *Jockey-3* ORF2 RNA quantification, the presence of *Jockey-3* RNA signal in the corresponding RNA-FISH images was determined by eye and recorded as present or absent. To determine if lacO transcripts were present, lacO RNA signal was determined by eye and recorded as present or absent. We selected metaphases with intact chromosome 3’s (identified with *dodeca* DNA-FISH) and with lacO at the 3peri location (identified with lacO DNA-FISH) for quantification.

### Fluorescence intensity quantifications

To measure *Jockey-3* signal at the centromeres of metaphase chromosomes, non-deconvolved in-focus z slices were quick-projected using the max intensity setting in SoftWorx. Polygons were drawn around the centromere of each chromosome using the edit polygons tool in the CENP-C channel then propagated to the *Jockey-3* channel to capture *Jockey-3* RNA max intensity fluorescence at the centromere. Similar polygons were used to capture background fluorescence for downstream calculations. Signal for sister centromeres were averaged and the average max intensity of the background fluorescence for that channel was subtracted. The measured max intensities for CENP-C and *Jockey-3* were plotted using Prism and compared.

For the quantification of metaphase spreads from sh-*Jockey-3* knockdowns, non-deconvolved 100x images were quick-projected in Softworks using the average intensity setting. Images were exported as TIFF and quantified with FIJI. In FIJI, a 400×400 pixel area including CENP-C, *Jockey-3* ORF1, and *Jockey-3* ORF2 foci on centromere Y was drawn to measure total intensities. Background intensities were set as lowest intensities in the square. Final fluorescence intensities in arbitrary units were calculated by subtracting background intensities from total intensities.

For the quantification of interphase spreads from sh-*Jockey-3* knockdowns, images were quick-projected in Softworks using the max intensity setting. Images were exported as TIFF and quantified with FIJI. In FIJI, entire nuclei were circled to measure raw max intensities of CENP-C, *Jockey-3* ORF1, and *Jockey-3* ORF2. Circles were then moved to the background area to measure background intensities. Final fluorescence intensities in arbitrary units were determined by subtracting background intensities from max intensities.

For the quantification of metaphase spreads from CAL1-GFP-LacI, *lacO*80C4 and GFP-LacI, *lacO*80C4, non-deconvolved 100x images were quick-projected in Softworks using the maximum intensity setting. Images were exported as TIFF and quantified with FIJI. In FIJI, a 400×400 pixel area including *lacO* foci on chromosome 3 was drawn to measure the total intensity. The background intensity was set as the average of 8 surrounding 400×400 pixel areas. The final fluorescence intensity in arbitrary units was calculated by subtracting the background intensity from the total intensity.

### Mapping *Jockey-3* RNA FISH probes to centromeres

To determine how many probes are predicted to bind to each centromere, we mapped probes to the centromeric contigs extracted from the heterochromatin-enriched genome assembly from (Chang et al., 2019) using the map to reference tool in Geneious, using all default settings and allowing all best matches.

### Embryo collection, RNA extraction, and nuclei isolation for PRO-seq

Embryos were collected from 2–3 days old iso-1 flies at 25°C. Adult flies were kept in multiple cages on grape juice agar plates containing a small amount of fresh yeast paste. Collection plates from the first 1h were discarded and flies were allowed to lay embryos on grape juice agar plates for 12 hrs overnight. Embryos were rinsed thoroughly with water and egg wash (0.7% NaCl made in DEPC treated water plus 0.05% Triton-X 100) in a mesh basket. Embryos were then dechorionated with 50% bleach for 1 minute, rinsed thoroughly with tap water in a mesh basket, flash-frozen in liquid nitrogen, and stored at −80°C.

For RNA-seq, frozen embryos were resuspended in 300μl of TRI Reagent (Sigma Aldrich T9424) and homogenized using a motorized pestle. After centrifugation, RNA was extracted from the supernatant using the Zymo DirectZOL kit (Zymo Research) following the manufacturer’s instructions.

Embryo nuclei isolation was performed largely as described in (Saunders et al., 2013). 50–100μl packed embryos were resuspended in 1mL cold buffer 1 (1M sucrose, 1M Tris pH 7.5, 1M MgCl_2_, 100% Triton X-100, 100mM EGTA, 1M DTT, 1x PTase inhibitor cocktail Roche, 20U/μl SUPERase In Ambion, 1M CaCl_2_), dounced in a 1m dounce homogenizer with a loose pestle 25 times, centrifuged at 900g for 2 min at 4°C to remove large debris, and dounced again with a tight pestle 15 times on ice. Nuclei were pelleted at 800g for 10 min at 4°C and washed twice in buffer 1 and once in freezing buffer (1M Tris pH 8, 100% glycerol, 100mM MgAc_2_, 0.5M EDTA, 1M DTT, 1x PTase inhibitor cocktail Roche, 20U/μl SUPERase In Ambion). Nuclei were resuspended in freezing buffer, flash-frozen, and stored at −80°C until use.

### Nuclei and RNA isolation from larval brains for PRO-seq and RNA-seq

Crawling larvae (3rd instar) were washed and dissected in PBS. Approximately 125 brains were dissected, flash frozen in liquid nitrogen, and stored at −80°C. Nuclei isolation was performed as described for the embryos but using a 0.5ml dounce homogenizer. Total RNA extraction was performed as described for embryos.

### PRO-seq library generation, pre-processing and alignment

PRO-seq libraries were prepared as previously described (Mahat et al., 2016). 0.9–4.5 × 10^6^ nuclei were mixed with permeabilized 1 × 10^6^ Hela nuclei (as spike-in) in 4-biotin-NTP run-on reactions. Run-on RNA was then base-hydrolyzed for 20 min on ice and enriched using M280 streptavidin beads and TRIzol extraction. After amplification, libraries were purified by polyacrylamide gel electrophoresis (PAGE) to remove adapter-dimers and to select molecules below 650 bp in size. Libraries were then sequenced on an Illumina NextSeq 500/550, producing paired-end 100bp reads. We obtained around 71 million reads.

Raw fastq files were first trimmed for quality (q 20), length (20 bp), and adapter sequences removed using cutadapt(Martin, 2011). For use with Bowtie 2 (Langmead and Salzberg, 2012), paired-end reads were aligned to a combined Human (GRCh38) - *Drosophila* heterochromatin-enriched assembly (Chang et al., 2019) using default “best match” parameters. For use with Bowtie, read 1 was reverse-complemented using the fastx-toolkit (http://hannonlab.cshl.edu/fastx_toolkit) and then aligned to a combined Human (GRCh38) - *Drosophila* heterochromatin-enriched assembly using k-100 parameters (reporting up to 100 mapped loci for each read). In each case, sorted bam files containing reads mapping to *Drosophila* were processed into bed files using BEDtools (Quinlan and Hall, 2010), which were used for either: 1) unique 21-mer filtering (described below in “Meryl unique k-mer filtering”), or 2) generation of 3’ end only (RNA polymerase occupancy position) bed files. In the case of option 2) these 3’ end only bed files were then use for either: 1) counting read abundance and coverage with BEDtools, or 2) BigWig file generation for visualization in the Integrated Genome Viewer (IGV) (Robinson et al., 2011).

### RNA-seq library generation, pre-processing, and alignment

RNA-seq libraries were generated from 0–12h embryos and 3rd instar larval brains using Illumina stranded total RNA prep, with the ligation performed with Ribo-Zero Plus and sequenced on Illumina TruSeq Stranded total RNA library prep kit, producing 150bp paired-end reads. We obtained around 46 million reads.

Raw fastq files were first trimmed for quality (q 20) and length (100 bp), and then adapter sequences removed using cutadapt (Martin, 2011) before being aligned to a *Drosophila* heterochromatin-enriched assembly (Chang et al., 2019) as paired-end reads using either Bowtie 2 (Langmead and Salzberg, 2012) default “best match” parameters or Bowtie k-100 (Langmead et al., 2009). HeLa spike-ins were not included in RNA-seq data and therefore, did not need to be removed. In each case, sorted bam files were processed into bed files using BEDtools (Quinlan and Hall, 2010), which were used for one of the following: 1) unique 51-mer filtering, 2) counting read abundance and coverage with BEDtools, or 3) BigWig file generation (BEDtools, GenomeBrowser/20180626) for visualization in the Integrated Genome Viewer (IGV) (Robinson et al., 2011).

### Meryl unique k-mer filtering

Single copy k-mers were generated from *Drosophila* heterochromatin-enriched assembly using Meryl (Rhie et al., 2020). We chose the length of single-copy k-mers (21 versus 51-mers) to use for filtering based on the length of the library insert, which is smaller for PRO-seq than for RNA-seq. Bed files of the mapped reads were used to filter through Meryl single copy k-mers using overlapSelect with the option ‘-overlapBases=XXbp’ (XX represents the length of the single copy k-mers (21-mer or 51-mer); GenomeBrowser/20180626). This locus-level filtering requires a minimum of the entire length of k-mer should overlap with a given read in order to be retained. The bed files from all RNA-seq mapping methods (default, k-100, and k-100 51-mer filtered) were used for read counts for repeats and BigWig file generation of IGV visualization (Robinson et al., 2011). The bed files from all PRO-seq mapping methods (default, k-100, and k-100 21-mer filtered) were first processed into 3’ end only (RNA polymerase occupancy position) bed files before being used for read counts across repeats and BigWig file generation for IGV visualization.

### Centromere heat maps for PRO-seq and RNA-seq data

The density of all centromeric repeats was obtained by counting the number of reads mapping to each repeat and dividing it by the number of total reads mapping to that centromeric contig . Read counts of all repeats were obtained with bedtools coverage -counts option. All heatmaps were generated with the ggplot2 R package.

### CUT&Tag from embryos

2–12h old *Drosophila* iso-1 embryos were collected from cages containing grape-juice agar plates with yeast paste incubated overnight at 25°C. Embryos were washed in embryo wash buffer (0.7% NaCl, 0.04% Triton-X100) and then were dechorionated with 50% bleach for 30s. Embryos were lysed in 1ml buffer B (pH7.5, 15mM Tris-HCl, 15mM NaCl, 60mM KCl, 0.34M Sucrose, 0.5mM Spermidine, 0.1% β-mercaptoethanol, 0.25mM PMSF, 2mM EDTA, 0.5mM EGTA) using a homogenizer and filtered through a mesh to remove large debris. Nuclei were spun at 5000g for 5 min and resuspended in 500μl of buffer A (pH7.5, 15mM Tris-HCl, 15mM NaCl, 60mM KCl, 0.34M Sucrose, 0.5mM Spermidine, 0.1% β-mercaptoethanol, 0.25mM PMSF) twice. The final pellet was resuspended in CUT&Tag wash buffer (20mM HEPES pH 7.5, 150mM NaCl, 0.5 mM Spermidine) to a final concentration of 1 million nuclei/ml.

CUT&Tag was performed on approximately 50,000 nuclei per sample using the pA-Tn5 enzyme from Epycpher, following the manufacturer’s instructions (CUT&Tag Protocol v1.5; (Kaya-Okur et al., 2019). We used a rabbit anti-Cid/CENP-A antibody (Active Motif cat. 39713, 1:50) and rabbit anti-IgG as negative control (1:100). For the library preparation, we used the primers from (Buenrostro et al., 2015). Before final sequencing, we pooled 2μl of each library and performed a MiSeq run. We used the number of resulting reads from each library to estimate the relative concentration of each library and ensure an equal representation of each library in the final pool for sequencing. We sequenced the libraries in 150-bp paired-end mode on HiSeq Illumina. We obtained around 6–9 million reads per library, except for the IgG negative control which typically yields much lower reads.

### CUT&Tag mapping

Raw fastq files of CUT&Tag data were trimmed using trimgalore with these options --paired --nextera --length 35 --phred33 and read quality was assessed with FASTQC. Reads were mapped to Drosophila heterochromatin-enriched assembly with Bowtie2. And MACS2 callpeak was used to call peaks using the IgG as our input control (options -c IgG.bam -f BAMPE -g dm -q 0.01 -B --callsummits). The CENP-A domains were defined based on MACS2 peaks and deepTools bamCompare (Ramirez et al., 2016) read coverage. The CENP-A domain for each centromere was determined from the first to the last MACS2 peak. Non-centromeric CENP-A domains were defined based on MACS2 peaks alone without having a single domain for each contig as compared to centromeres. As per Fig. 5B, MACS2 signal intensity values were averaged (BEDtools map -o mean; (Quinlan and Hall, 2010)) from the narrowPeak file across each CENP-A domain.

### Statistical tests

All *Jockey-3* sequences were extracted from *Drosophila* heterochromatin-enriched assembly annotations using BEDtools (Quinlan and Hall, 2010) and labeled as CENP-A-CEN, CENP-A-nonCEN, or nonCENP-A (requiring at least 1bp overlap with MACS2 CENP-A domains) using BEDtools map -o collapse. *Jockey-3* copies were also labeled as either full-length (FL; if containing a full ORF2) or truncated. For generation of nonCENP-A/nonCEN associated random genomic intervals for Fig. 5C, BEDtools random was used with options -l 773 (average size of a CENP-A-nonCEN domain) and -n 333 (equivalent to number of CENP-A-nonCEN domains). PRO-seq read counts were obtained with BEDtools coverage -counts (requiring at least 1bp overlap) for all *Jockey-3* copies in the genome, as well as for each CENP-A domain and CENP-A-nonCEN-sized random interval. Unique 21-mer coverage per *Jockey-3*, as well as *Jockey-3* coverage per CENP-A domain was assessed using BEDtools coverage. Unpaired *t* tests were performed to quantify differences and determine significance. Scatter box plots and bar graphs were generated via GraphPad Prism (v10.1.1).

### Code

All codes for analyses and figures are available on Github https://github.com/bmellone/Dmel-Centromere-Transcription

## Supplementary Material

Supplement 1 **Figure S1: PRO-seq reads aligned to genes show expected enrichment of RNA polymerase occupancy at gene promoters.** Heatmaps of RNA polymerase occupancy using Bowtie 2 default “best match” for antisense (blue) and sense (red) strands per gene. Averaged profiles (line graphs) across all genes are shown along the top including standard error shading (gray). All genes are anchored to the 5’ end (transcription start site (TSS)) with a specified distance into the gene body denoted in the bottom right (5kb), and a specified distance away from the gene body denoted in the bottom left (0.5kb). The dotted line per heatmap denotes the static end of each gene as the are included longest to shortest form top to bottom.
Fig. S2: FL vs truncated k-100 and k-100 filtered PRO-seq
**A** PRO-seq read density scatter boxplot comparisons between full-length (FL) and truncated *Jockey-3* copies, regardless of genome location. Mapping was done with Bowtie k-100 and k-100 21-mer filtered using single-end reads. Unpaired t-tests (Student’s t-test) were performed indicating a significant difference (****, p < 0.0001) between each group illustrating a consistent trend seen across all three mapping methods (Fig. 1B). Standard deviation error bars are shown.
**B** Meryl unique 21-mer coverage for FL and truncated *Jockey-3* copies. An unpaired t test (Student’s t-test) was performed indicating a significant difference (****, p < 0.0001), wherein truncated copies have more unique 21-mers as a result of having accumulated more mutations over time making them less similar to each other.
**Fig. S3: PRO-seq and RNA-seq from larval brains.** PRO-seq, RNA-seq signals for 3rd instar larval brains across all *D. melanogaster* centromeres. Top track shows sense, bottom, antisense. Tracks show read coverage with three mapping methods: Bowtie 2 default best match (“lower bounds”; yellow), over-fit (“upper bounds”; gray) and a filtered over-fit (“medium bounds”; blue). For PRO-seq we Bowtie1 k-100 for over-fit, and Bowtie1 k-100 21-mer filtered for medium bounds. For RNA-seq we used Bowtie2 k-100 for over-fit and Bowtie2 k-100 51-mer filtered for medium bounds. Repeat annotation is shown on top (see legend for details), with unique 21 and 51-mers (black) used for the filtering shown below. The k-mer tracks illustrate the regions that lack sequence specificity and are therefore most prone to read loss through k-mer filtering. Coordinates shown are kilobases. Dotted red line indicates the boundaries of the islands.
**Figure S4: RNA-FISH detects the chromosome-associated non-coding RNA *Rox1*.** RNA-FISH/IF on *D. melanogaster* (iso-1) mitotic chromosomes from male larval brains with the ORF2 of *Jockey-3* probe (yellow), a *Rox1* probe (cyan), and with CENP-C antibodies (green). DNA is stained with DAPI (magenta). Bars 1μm. Arrow points to *Rox1* (yellow) localization on the arms of the X chromosome.
**Figure S5: *Jockey-3* RNA localization on centromeres of individual chromosomes.** RNA-FISH/IF on *D. melanogaster* (iso-1) mitotic chromosomes from male larval brains. Individual chromosomes showing centromeric signal for each *Jockey-3* probe set (ORF2, ORF2 anti, and ORF1) (yellow) on chromosomes (X, Y, 2, 3, and 4), CENP-C showing the centromere (green), and DAPI (magenta). Insets show CENP-C and *Jockey-3* signals. Bars 1μm
Figure S6 *Jockey-3* RNA localizes to mitotic centromeres in other tissues and in *D. simulans*
**A** RNA-FISH/IF on mitotic chromosomes from *D. melanogaster* (iso-1) adult ovaries. IF for CENP-C (green) and RNA-FISH for *Jockey-3* ORF2 (yellow). DNA is stained with DAPI (magenta).
**B** RNA-FISH/IF on mitotic spreads from S2 cells. IF for CENP-C (green), and RNA-FISH for *Jockey-3* ORF2 (yellow) and SatIII (found on X and 3rd chromosomes; cyan). DNA is stained with DAPI (magenta).
**C** RNA-FISH/IF on *D. simulans* (laboratory stock w501) mitotic chromosomes from male larval brains. IF with CENP-C (green) and RNA-FISH for *Jockey-3* ORF2 and *Rox1* (stains the X, control; cyan). DNA is stained with DAPI (magenta). Bar 1μm
Figure S7: RNA foci detected with *Jockey-3* ORF2 correspond to RNA, not DNA
**A** RNA-FISH on *D. melanogaster* (iso-1) mitotic chromosomes from male larval brains for the 3’ sense of *Jockey-3* (yellow) and an OligoPaint for 61C7 (green). Green arrow indicates presence of signal, white arrow indicates lack of signal.
**B** DNA-FISH on *D. melanogaster* (iso-1) mitotic chromosomes from male larval brains with stellaris FISH probes for ORF2 of *Jockey-3* (yellow) and an OligoPaint for 61C7 (green). Green arrow indicates presence of signal, white arrow indicates lack of signal. Bars 5μm.
Figure S8: RNAse treatments result in a decrease in *Jockey-3* RNA-FISH signal
**A** RNA-FISH with *Jockey-3* ORF2 on *D. melanogaster* male larval brain monolayers expressing eCENP-A-EGFP (green). DNA is stained with DAPI (magenta). Shown are the before and After treatments with and without RNaseH.
**B** Quantification of the number of ORF2 *Jockey-3* foci before and After RNaseH treatment (not significant for before and p<0.0001 for After treatment). Quantification of the number of eCENP-A-EGFP foci before and After RNaseH treatment (p=0.367 for before and not significant for After treatment). N=1 brain, n=107 cells quantified for before treatment and n=86 cells for After treatment).
**C** RNA-FISH with *Jockey-3* ORF2 and *Rox1* (cyan; control) on *D. melanogaster* male larval brain monolayers expressing eCENP-A-EGFP (under the endogenous CENP-A promoter; green).
**D** Quantification of eCENP-A-GFP (N=1 brain, n=100 cells; *p = 0.0292*)* and ORF2 *Jockey-3* foci (N=1 brain, n=100 cells; ****p = <0.0001).
**Figure S9: Quantification of non-centromeric *Jockey-3* foci in mitotic cells.** Graph showing the non-centromeric localization of *Jockey-3* (ORF2, ORF2 anti, and ORF1) on mitotic chromosomes from larval brain squashes. XR, 4L, and 4R were not quantified since these arms are cytologically too small and too close to the centromeres to be distinguished. ORF2 (N=3 brains, n=83 spreads), ORF2 anti (N=3 brains, n=28 spreads), and ORF1 (N=4 brains, n=69 spreads).
Figure S10: RNA-FISH *Jockey-3* foci are present during interphase
**A** RNA-FISH/IF with ORF2 (yellow) and ORF1 (magenta) *Jockey-3* probes and CENP-C (green) on interphase cells from male larval brain squashes.
**B** IF/RNA-FISH as in A on S2 cells (30% of cells have at least 1 co-localizing CENP-C/*Jockey-3* spot, n=54; note that S2 cells do not have a Y chromosome).
**C** RNA-FISH/IF as in A on ovary squashes. Insets show magnification of centromeres in the box.
**D** Graph showing the average number of *Jockey-3* ORF2 foci that co-localize with CENP-C (cen) versus not (non-cen).
**E** Graph of the average number of centromeric *Jockey-3* foci in interphase versus metaphase cells. N=3 brains, n=30–105 cells per brain.
**F** Graph showing the % of cells showing 2 or more *Jockey-3* foci co-localizing with CENP-C in interphase versus mitosis. Data in **D-F** is all from the same 3 male larval brains as in **A**.
**Figure S11: RNA-FISH for centromeric retroelement *Doc*.** RNA-FISH/IF on *D. melanogaster* (iso-1) male larval brain squashes. Immunofluorescence for CENP-C (green), and RNA-FISH for *Jockey-3* ORF2 (yellow) and *DOC* sense (cyan). *Doc* is present in the islands of centromere X and 4. Dashed box shows the X centromere lacking *Doc* signal. The solid line box shows a centromere with *Doc* signal in interphase. DNA is stained with DAPI (magenta).
**Figure S12: IGV tracks for CENP-A CUT&Tag.** IGV tracks showing CUT&Tag signals for 0–12h embryos across all *D. melanogaster* centromeres. Top track shows color-coded repeat annotation (details in legend). CUT&Tag track shows CENP-A enrichment in gray. Red dotted line shows the span of the CENP-A domain we defined for each centromere. Predicted MACS2 peaks for CUT&Tag data are shown in bottom track (black).
Figure S13: CENP-A associated k-100 and k-100 filtered PRO-seq.
**A** Bar graph illustrating the proportion of CENP-A associated *Jockey-3* copies expressed within the centromere and outside the centromere. PRO-seq mapping was done with Bowtie k-100 and k-100 21-mer filtered using single-end reads. Expression is defined as having either at least two PRO-seq read overlaps. The trend difference seen between Bowtie 2 default and Bowtie k-100 methods can be attributed to the lower unique 21-mer coverage of CENP-A copies allowing more reads to map to these copies.
**B** Meryl unique 21-mer coverage for CENP-A associated *Jockey-3* copies based on centromeric loci designation. Unpaired t tests (Student’s t-test) were performed indicating a significant difference (****, p < 0.0001; ***, p < 0.001; ns (non-significant), p > 0.05) between each group. Standard deviation error bars are shown.

Supplement 2 **Table S1: PRO-seq read and unique 21-mer coverage across all *Jockey-3* loci.** Table showing all 329 *Jockey-3* copies per CENP-A and centromeric association. PRO-seq read coverage for all three mapping methods are included: Bowtie 2 default “best match” using paired-end reads, and Bowtie k-100 and Bowtie k-100 21-mer filtered, both using single-end reads. Coverage of Meryl unique 21-mers per copy is also shown. Data included was used for Figs. 1B–C, Figs. 5G–H, Figs. S2 and S13.

Supplement 3 **Table S2: Read counts for PRO-seq.** Table showing the PRO-seq read count for each centromeric repeat within all centromere contigs. This data was used to generate the heatmaps shown in Fig. 1.

Supplement 4 **Table S3: *Jockey-3* RNA-FISH probe sequences mapped across the genome.** The table shows the chromosome, contig, and coordinates of every *Jockey-3* copy in the genome. The first tab shows just the full-length copies, the second shows all the centromeric and the last all non-centromeric insertions. Indicated are the type of chromatin they are found in (if known; designated as in (Chang et al., 2019)), approximate cytological location and number of probes predicted to bind. This information was used for the graph in Fig. 2F.

Supplement 5 **Table S4: CENP-A domain loci, both centromeric and non-centromeric.** Table showing all five centromeric and 333 non-centromeric CENP-A domains as defined by MACS2 peak calls from CUT&Tag data. Size (basepairs) and average MACS2 peak signal intensity shown per CENP-A domain. Data included was used for Figs. 5A–C.

Supplement 6 **Table S5: Proportion of *Jockey-3* copies expressed based on PRO-seq read overlap.** Table showing the number of *Jockey-3* copies (FL and truncated) expressed per CENP-A and centromeric association. Expression is defined as having at least two PRO-seq read overlaps. All three mapping methods are included: Bowtie 2 default “best match” using paired-end reads, and Bowtie k-100 and Bowtie k-100 21-mer filtered, both using single-end reads. Data included was used for Figs. 5D–E and Fig. S13.

Supplement 7 **Table S6: Summary of CENP-A associated truncated and full-length (FL) *Jockey-3* insertions.**Table showing the distribution of *Jockey-3* copies associated with CENP-A and/or centromeres across the genome. A column for other repeats, excluding *Jockey-3*, is shown to emphasize the enrichment of *Jockey-3* associated with CENP-A. Data included was used for Fig. 5F.

Supplement 8

## Figures and Tables

**Table 1:** Summary of the location of truncated and full-length (FL) *Jockey-3* insertions.

CEN	CEN_2	CEN_3	CEN_4	CEN_X	CEN_Y	CEN (all)	nonCEN
**All *Jockey-3***	2	11	21	21	147	202	127
**FL *Jockey-3***	0	0	0	0	9	9	14
**Truncated *Jockey-3***	2	11	21	21	138	193	113

**Table 2.** Centromere mapping of RNA-FISH probes.

RNA-FISH probes mapping	ORF2	ORF2 antisense	ORF1
**Cen X**	73	73	36
**Cen Y**	1117	1117	242
**Cen 2**	4	4	44
**Cen 3**	70	70	3
**Cen 4**	130	130	38

Table showing the total number of *Jockey-3* RNA-FISH probes predicted to bind to each centromeric contigs.

**Table 3.** Summary of the CENP-A domains and associated *Jockey-3* insertions.

CEN designation	# CENPA domains	# CENPA domains containing *Jockey-3*
**CEN**	5 (1/chromosome)	5 (100%)
**nonCEN**	333	43 (12.91%)
