# Supplementary material for: A centromere-derived retroelement RNA localizes in cis and is a core element of the transcriptional landscape of Drosophila centromeres
Source: bioRxiv. 2024 Jan 16:2024.01.14.574223. Preprint. [Version 2] doi: 10.1101/2024.01.14.574223 (PMC10827089; doi:10.1101/2024.01.14.574223)
Supplement: Supplement 1 — Figure S1: PRO-seq reads aligned to genes show expected enrichment of RNA polymerase occupancy at gene promoters. Heatmaps of RNA polymerase occupancy using Bowtie 2 default “best match” for antisense (blue) and sense (red) strands per gene. Averaged profiles (line graphs) across all genes are shown along the top including standard error shading (gray). All genes are anchored to the 5’ end (transcription start site (TSS)) with a specified distance into the gene body denoted in the bottom right (5kb), and a specified distance away from the gene body denoted in the bottom left (0.5kb). The dotted line per heatmap denotes the static end of each gene as the are included longest to shortest form top to bottom. Fig. S2: FL vs truncated k-100 and k-100 filtered PRO-seq A PRO-seq read density scatter boxplot comparisons between full-length (FL) and truncated Jockey-3 copies, regardless of genome location. Mapping was done with Bowtie k-100 and k-100 21-mer filtered using single-end reads. Unpaired t-tests (Student’s t-test) were performed indicating a significant difference (****, p < 0.0001) between each group illustrating a consistent trend seen across all three mapping methods (Fig. 1B). Standard deviation error bars are shown. B Meryl unique 21-mer coverage for FL and truncated Jockey-3 copies. An unpaired t test (Student’s t-test) was performed indicating a significant difference (****, p < 0.0001), wherein truncated copies have more unique 21-mers as a result of having accumulated more mutations over time making them less similar to each other. Fig. S3: PRO-seq and RNA-seq from larval brains. PRO-seq, RNA-seq signals for 3rd instar larval brains across all D. melanogaster centromeres. Top track shows sense, bottom, antisense. Tracks show read coverage with three mapping methods: Bowtie 2 default best match (“lower bounds”; yellow), over-fit (“upper bounds”; gray) and a filtered over-fit (“medium bounds”; blue). For PRO-seq we Bowtie1 k-100 for over-fit, and Bowtie1 k- [file media-1.pdf]

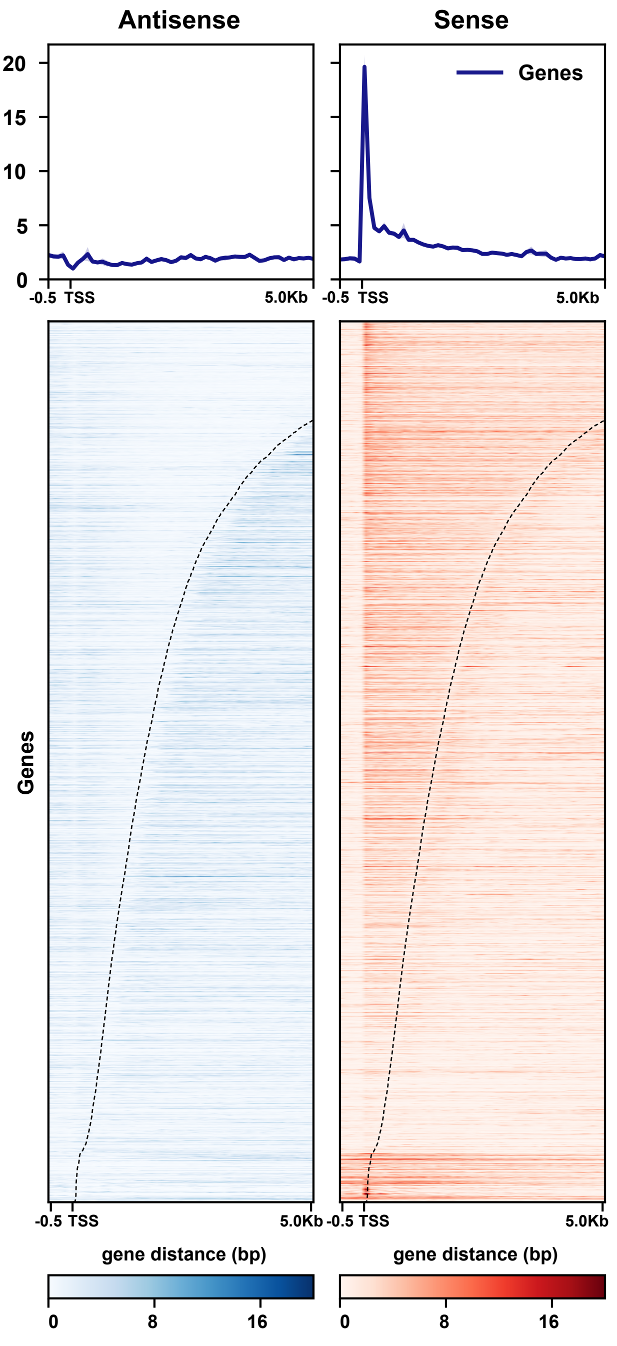
Antisense
Sense
Genes
20
15
10
5
0
-0.5
TSS
5.0Kb
Genes
gene distance (bp)
0
8
16

Fig.S1

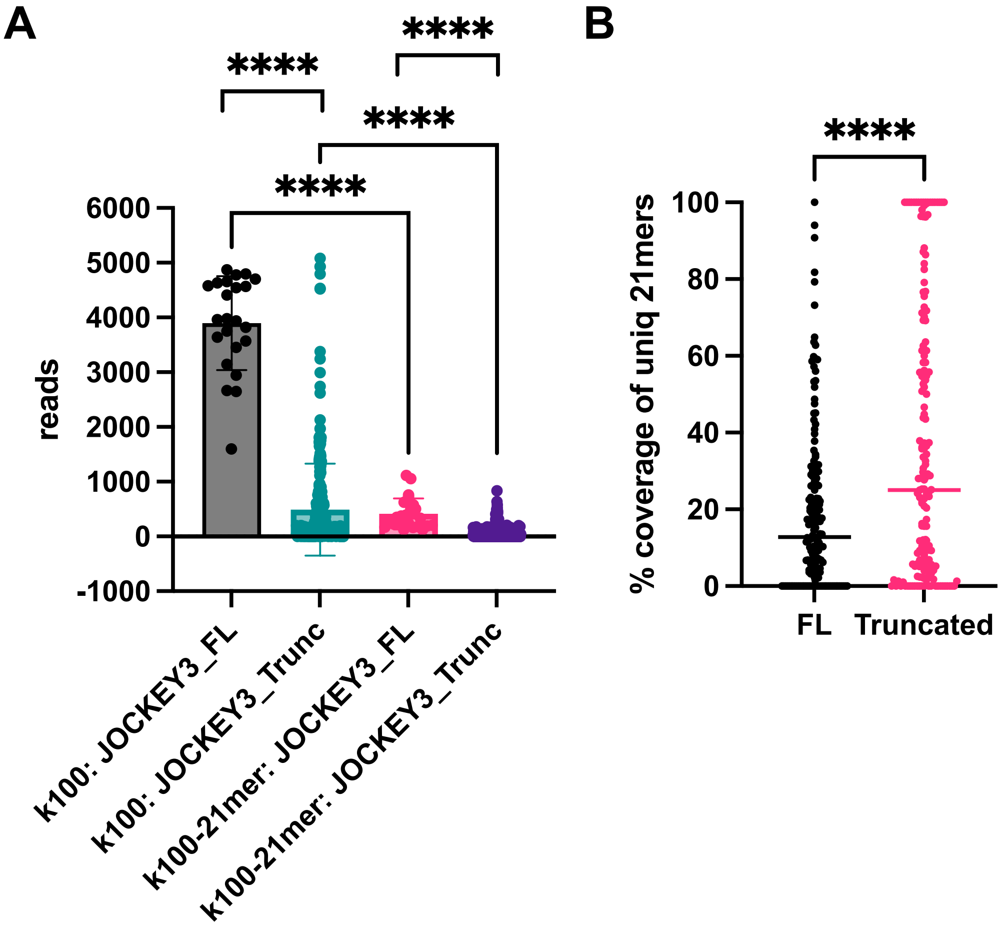
A
B
****
****
****
****
****
reads
6000
5000
4000
3000
2000
1000
0
-1000
k100: JOCKEY3_FL
k100: JOCKEY3_Trunc
k100-21mer: JOCKEY3_FL
k100-21mer: JOCKEY3_Trunc
% coverage of uniq 21mers
100
80
60
40
20
0
FL
Truncated

Fig. S2

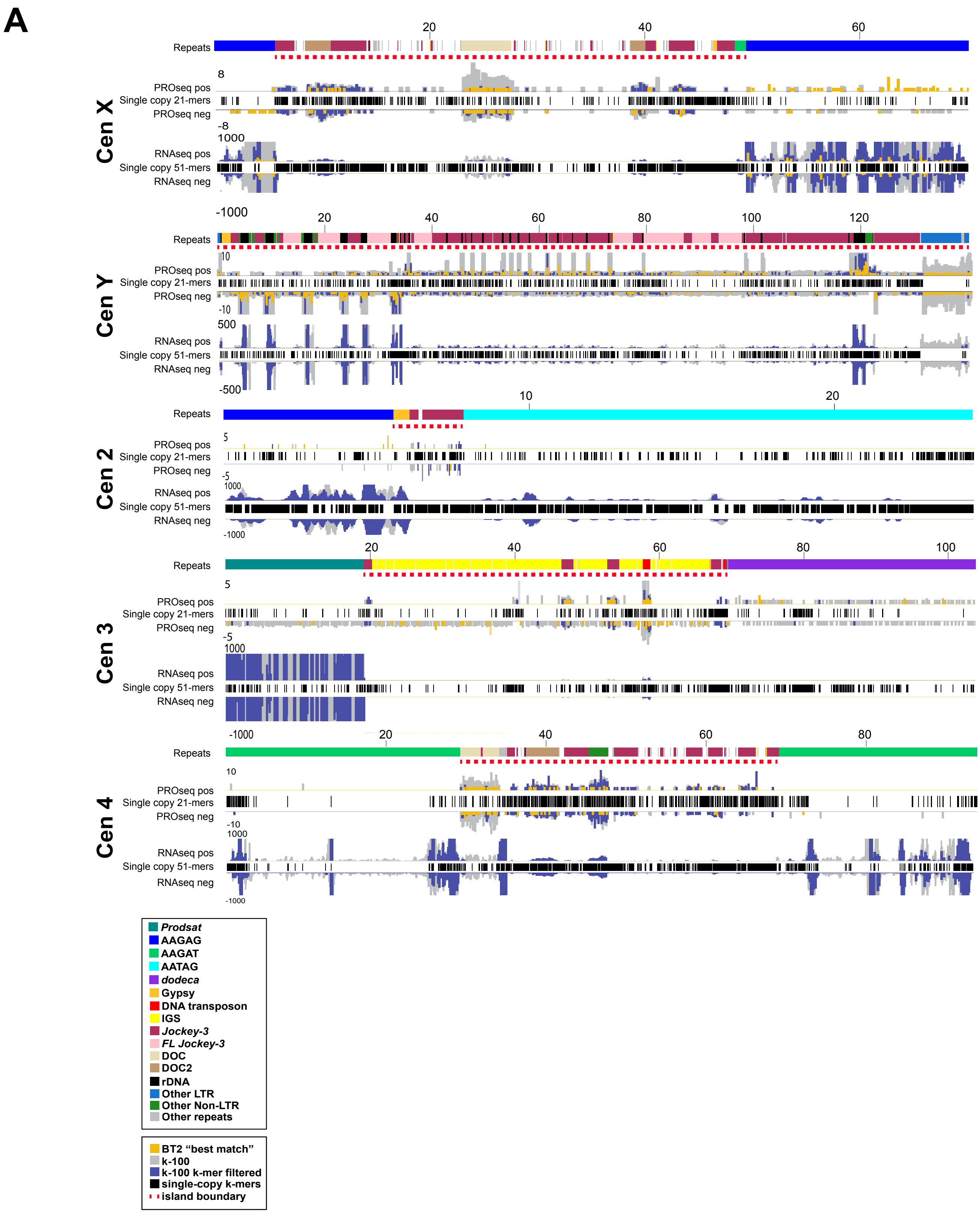
A
Cen X
Cen Y
Cen 2
Cen 3
Cen 4
Repeats
PROseq pos
Single copy 21-mers
PROseq neg
RNAseq pos
Single copy 51-mers
RNAseq neg
Prodsat
AAGAG
AAGAT
AATAG
dodeca
Gypsy
DNA transposon
IGS
Jockey-3
FL Jockey-3
DOC
DOC2
rDNA
Other LTR
Other Non-LTR
Other repeats
BT2 “best match”
k-100
k-100 k-mer filtered
single-copy k-mers
island boundary

Fig. S3

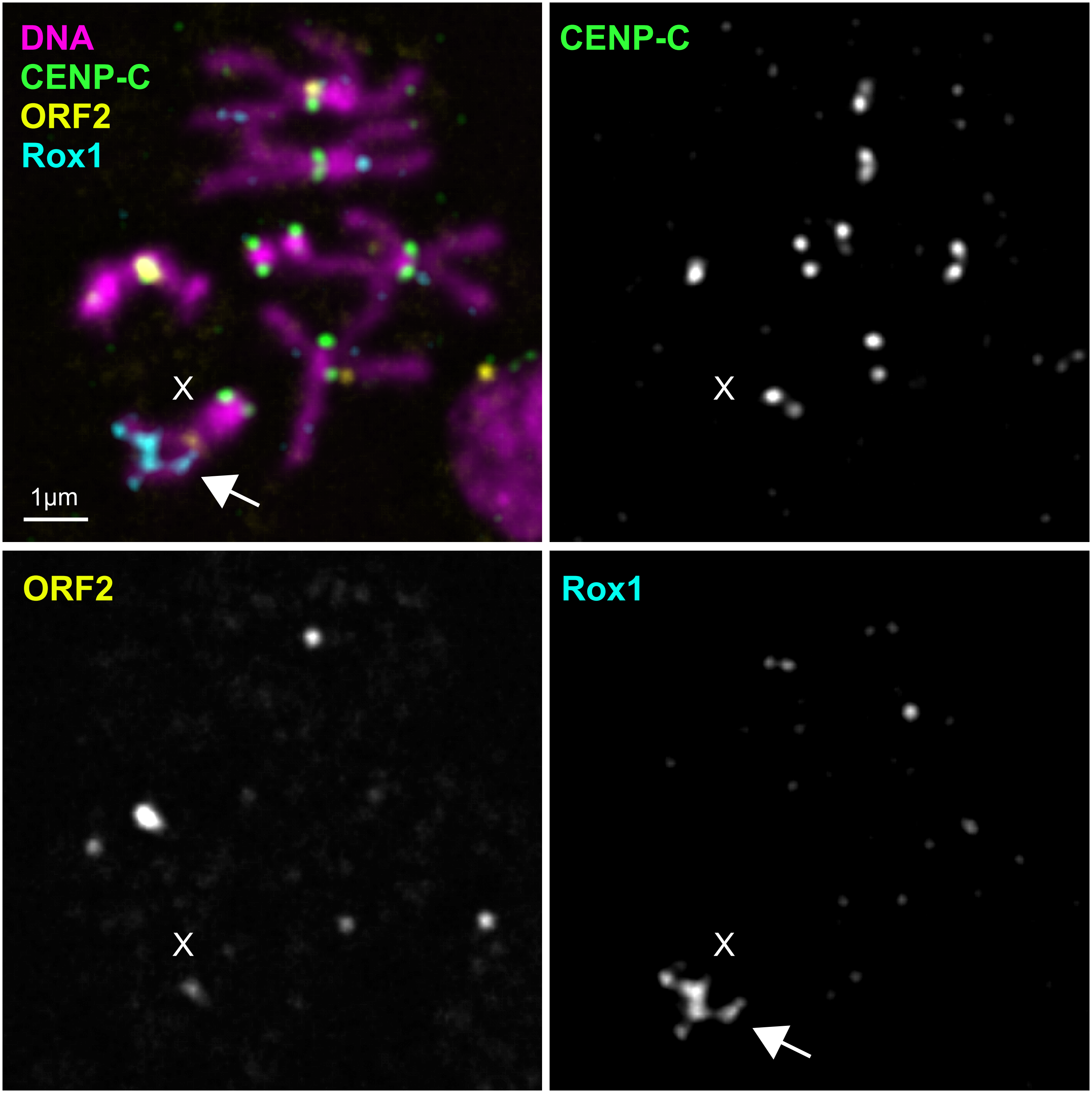

Fig. S4

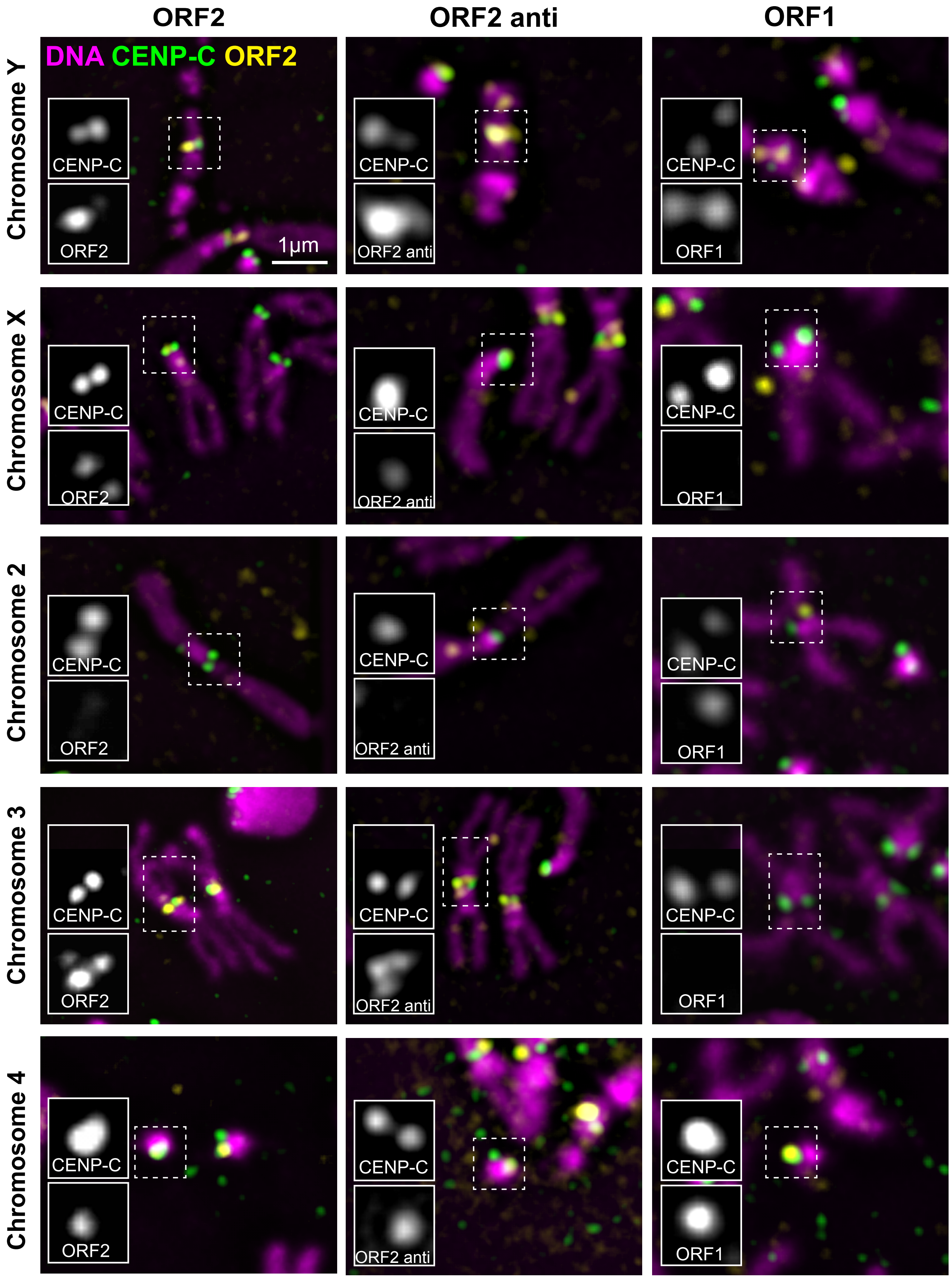
ORF2
ORF2 anti
ORF1
DNA CENP-C ORF2
Chromosome Y
Chromosome X
Chromosome 2
Chromosome 3
Chromosome 4
CENP-C
ORF2
ORF2 anti
ORF1
1μm

Fig. S5

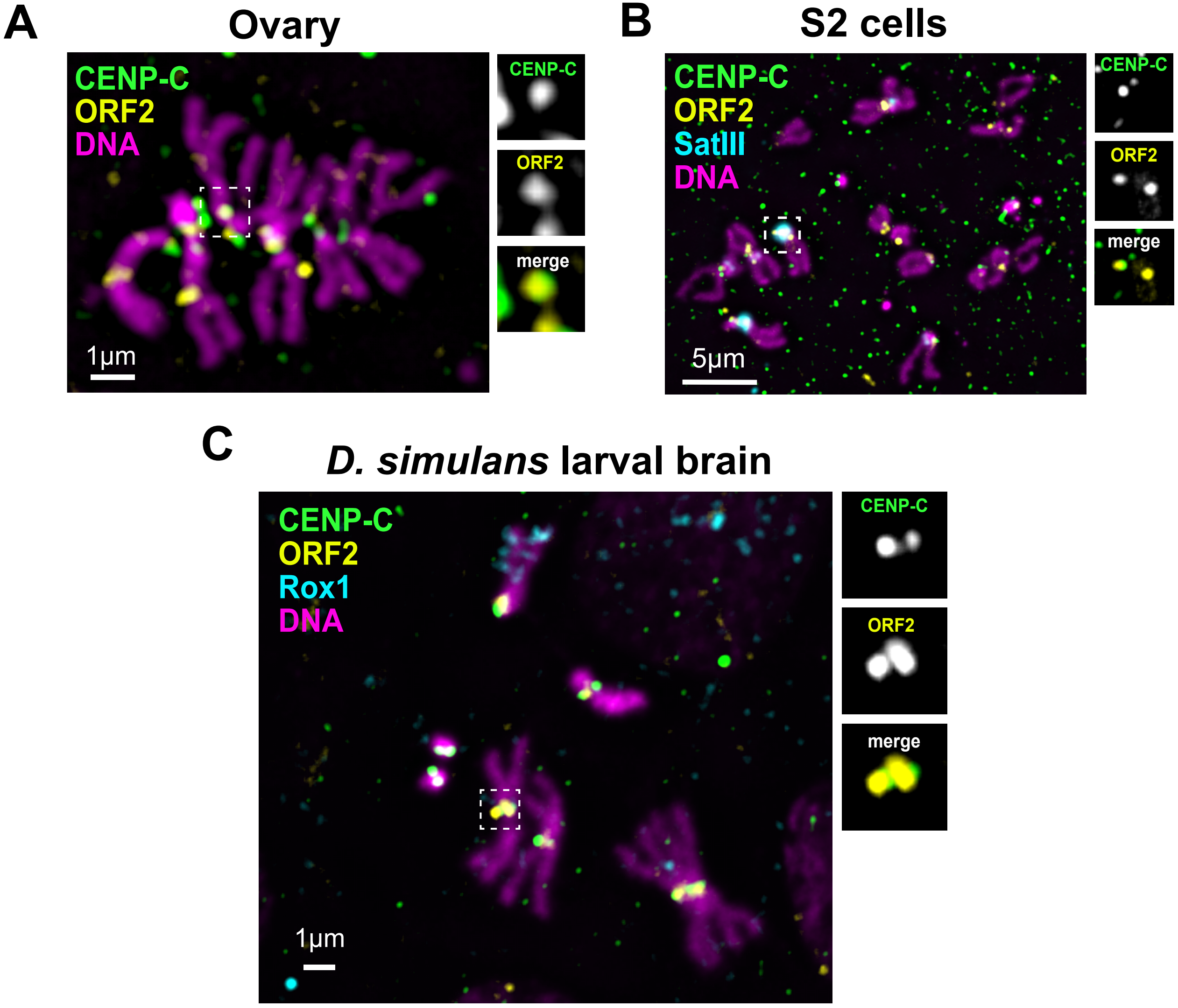
A
Ovary
CENP-C
ORF2
DNA
1µm
CENP-C
ORF2
merge
B
S2 cells
CENP-C
ORF2
SatIII
DNA
5µm
CENP-C
ORF2
merge
C
D. simulans larval brain
CENP-C
ORF2
Rox1
DNA
1µm
CENP-C
ORF2
merge

Fig. S6

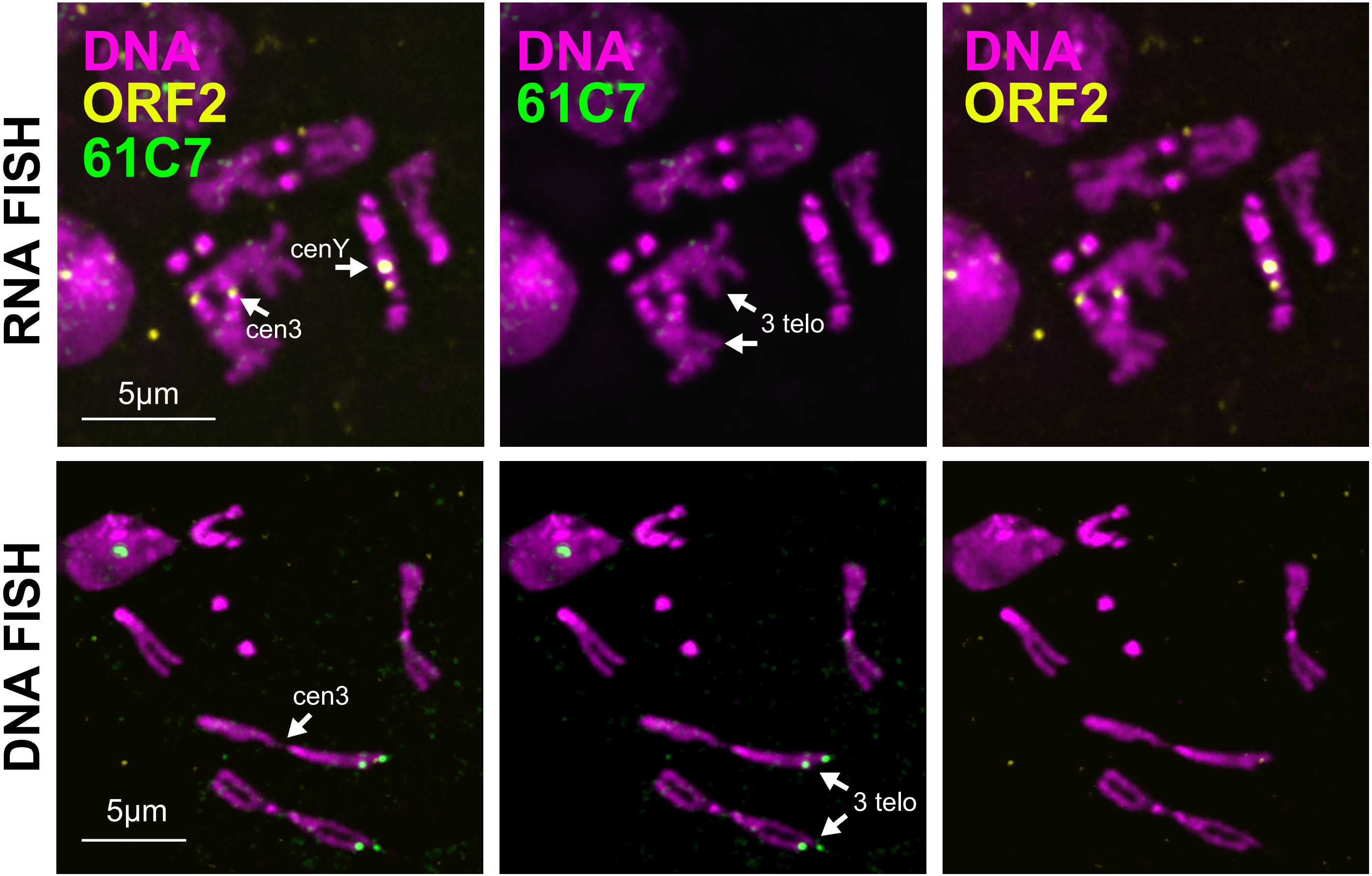

Fig. S7

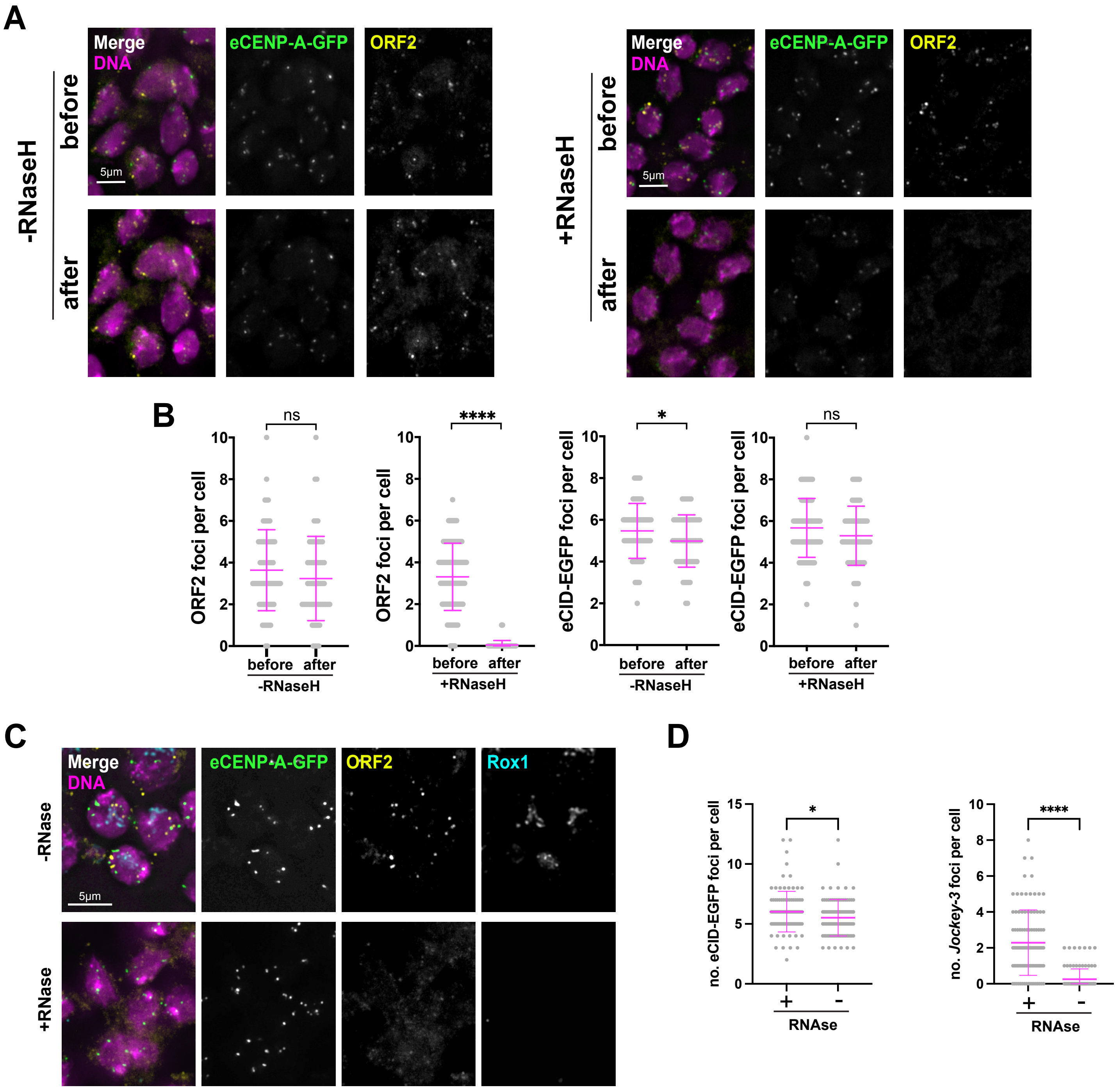
A
Merge
DNA
eCENP-A-GFP
ORF2
-RNaseH
before
after
5μm
+RNaseH
B
ns
****
*
ns
ORF2 foci per cell
eCID-EGFP foci per cell
before
after
-RNaseH
+RNaseH
C
Rox1
-RNase
+RNase
D
no. eCID-EGFP foci per cell
no. Jockey-3 foci per cell
RNAse
+
-

Fig. S8

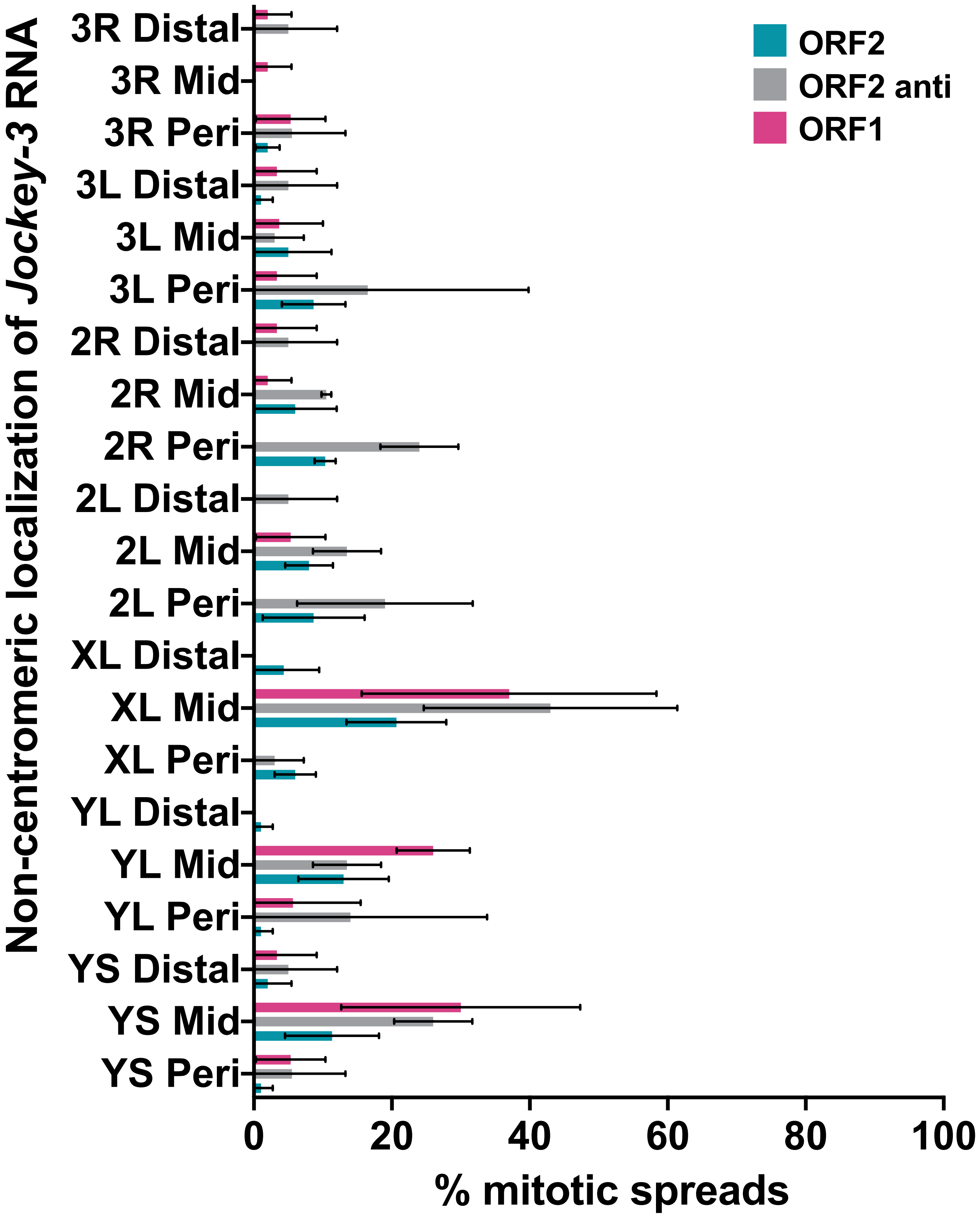

Non-centromeric localization of Jockey-3 RNA
3R Distal
3R Mid
3R Peri
3L Distal
3L Mid
3L Peri
2R Distal
2R Mid
2R Peri
2L Distal
2L Mid
2L Peri
XL Distal
XL Mid
XL Peri
YL Distal
YL Mid
YL Peri
YS Distal
YS Mid
YS Peri
ORF2
ORF2 anti
ORF1
0
20
40
60
80
100
% mitotic spreads

Fig. S9

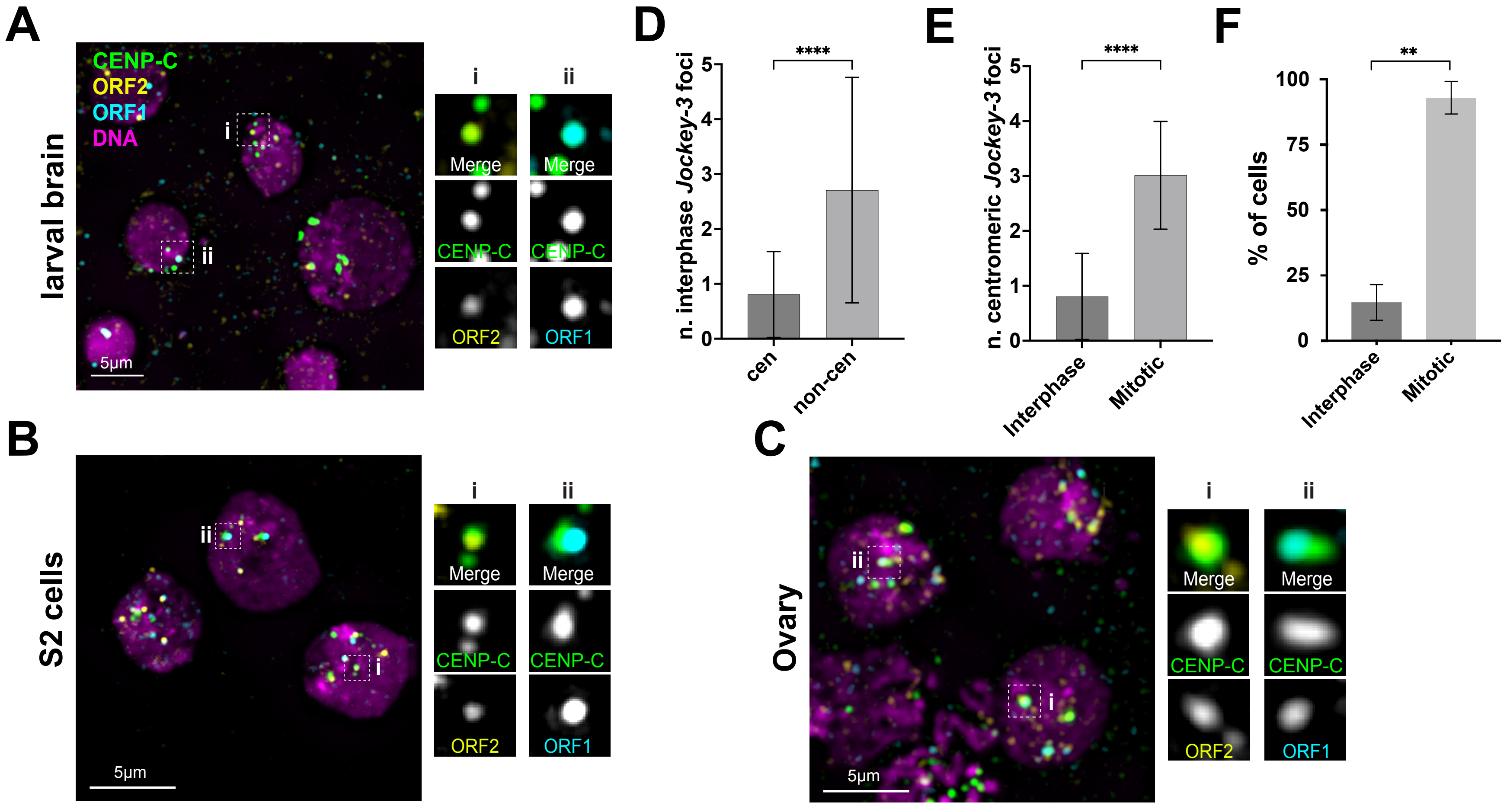
A
larval brain
CENP-C
ORF2
ORF1
DNA
i
ii
5μm
Merge
CENP-C
ORF2
ORF1
B
S2 cells
C
Ovary
D
n. interphase Jockey-3 foci
****
cen
non-cen
E
n. centromeric Jockey-3 foci
Interphase
Mitotic
F
% of cells
**
0
25
50
75
100

Fig. S10

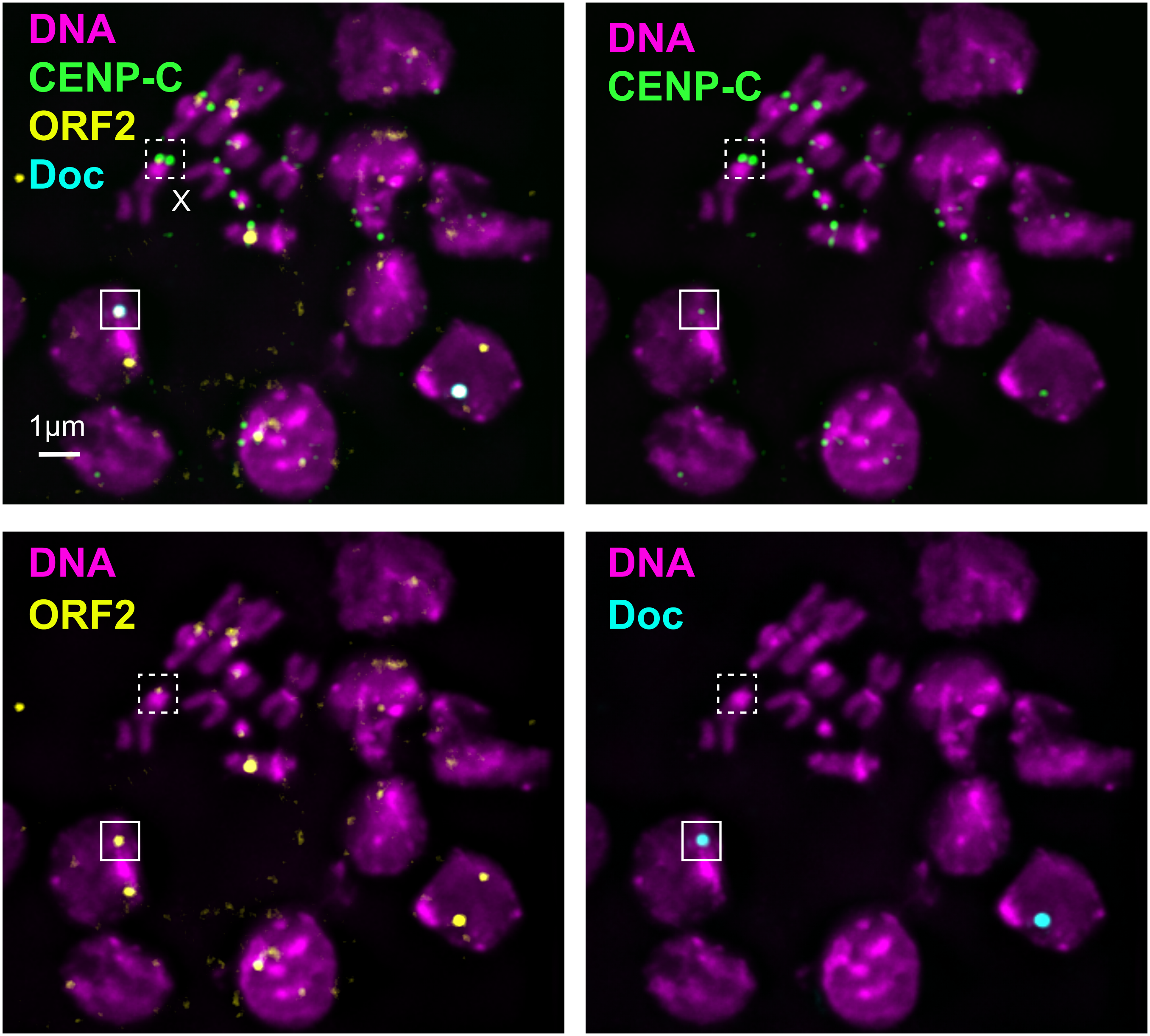

Fig. S11

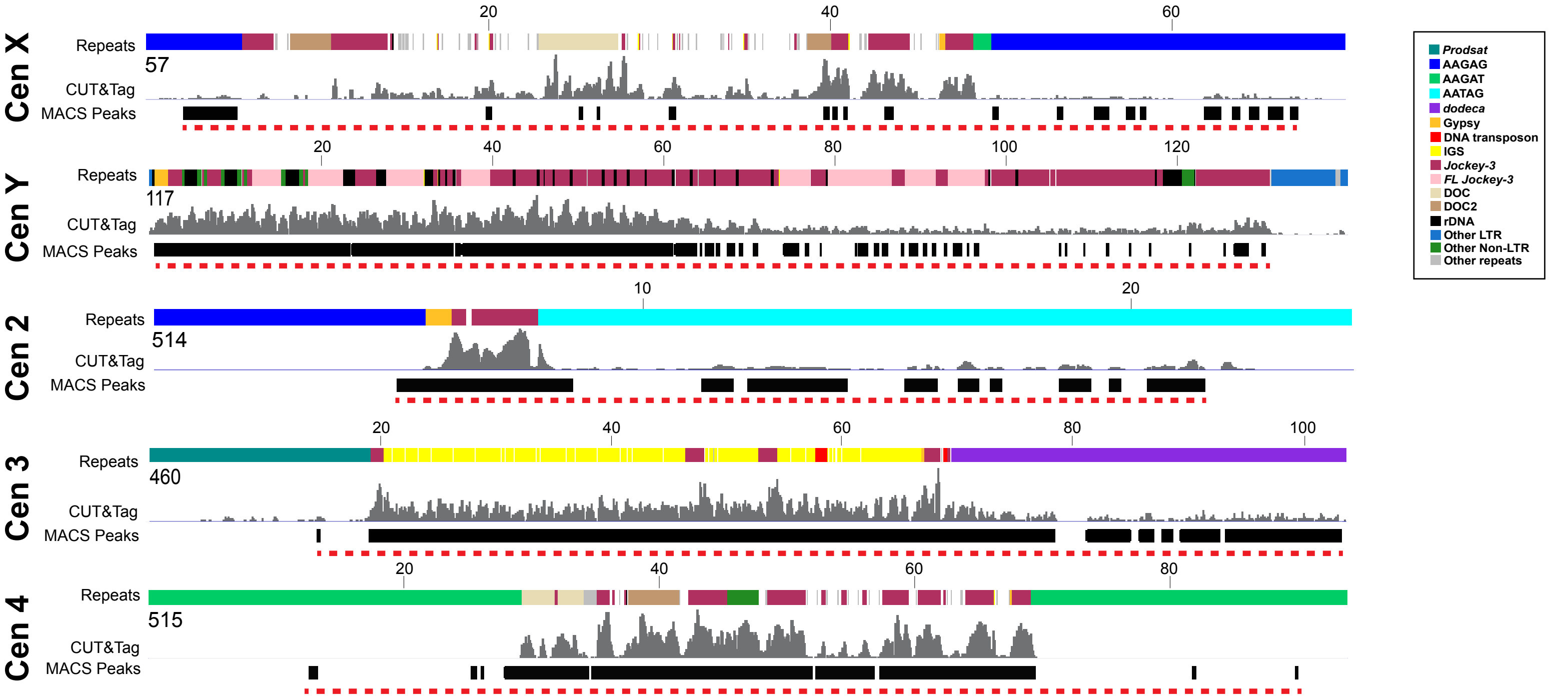
Cen X
Repeats
CUT&Tag
MACS Peaks
57
20
40
60
Cen Y
Repeats
CUT&Tag
MACS Peaks
117
20
40
60
80
100
120
Cen 2
Repeats
CUT&Tag
MACS Peaks
514
10
20
Cen 3
Repeats
CUT&Tag
MACS Peaks
460
20
40
60
80
100
Cen 4
Repeats
CUT&Tag
MACS Peaks
515
20
40
60
80
Prodsat
AAGAG
AAGAT
AATAG
dodeca
Gypsy
DNA transposon
IGS
Jockey-3
FL Jockey-3
DOC
DOC2
rDNA
Other LTR
Other Non-LTR
Other repeats

Fig. S12

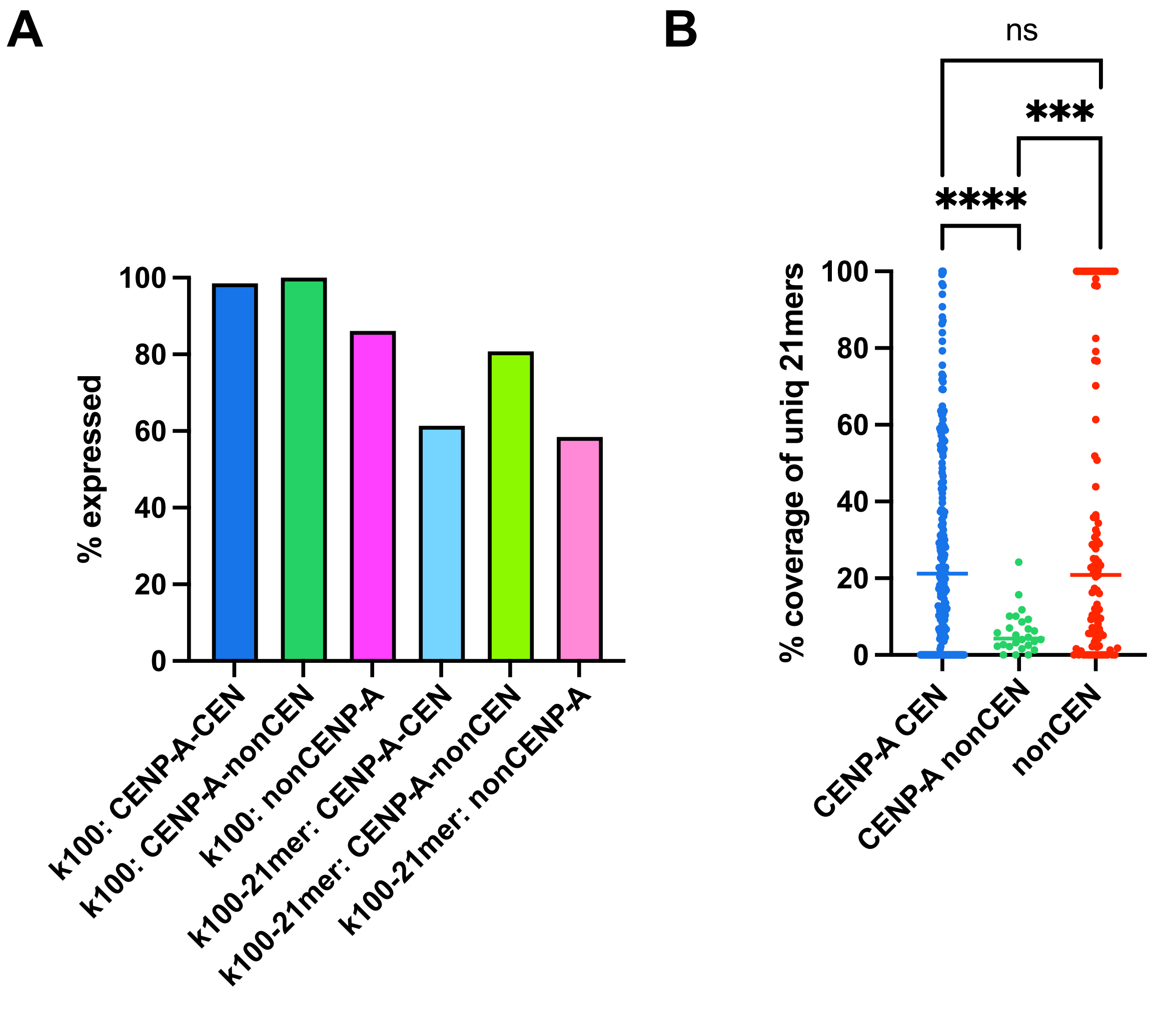
A
B
ns
***
****
100
80
60
40
20
0
% expressed
k100: CENP-A-CEN
k100: CENP-A-nonCEN
k100: nonCENP-A
k100-21mer: CENP-A-CEN
k100-21mer: CENP-A-nonCEN
k100-21mer: nonCENP-A
% coverage of uniq 21mers
CENP-A CEN
CENP-A nonCEN
nonCEN

Fig. S13
